# Association Between Folic Acid Use and Serum One-Carbon Metabolism-Related Metabolites in Maternal and Cord Blood of Japanese Pregnant Women

**DOI:** 10.3390/metabo16040215

**Published:** 2026-03-25

**Authors:** Yoshinori Kubo, Hideoki Fukuoka, Kumiko Shoji, Chisato Mori, Kenichi Sakurai, Midori Yamamoto, Masazumi Nishikawa, Kyoichi Oshida, Terue Kawabata

**Affiliations:** 1 Faculty of Nutrition, Kagawa Nutrition University, 3-9-21 Chiyoda, Sakado 350-0288, Saitama, Japan; shoji.kumiko@eiyo.ac.jp (K.S.); kawabata@eiyo.ac.jp (T.K.); 2 Division of Anatomy and Cell Biology, Department of Anatomy, Shiga University of Medical Science, Seta Tsukinowa-cho, Otsu 520-2192, Shiga, Japan; 3 Department of Nutrition and Metabolic Medicine, Center for Preventive Medical Sciences, Chiba University, 1-33 Yayoi-cho, Inage-ku, Chiba 263-8522, Chiba, Japan; hideoki.fukuoka@gmail.com (H.F.); sakuraik@faculty.chiba-u.jp (K.S.); 4 Department of Bioenvironmental Medicine, Graduate School of Medicine, Chiba University, 1-8-1 Inohana, Chuo-ku, Chiba 260-8670, Chiba, Japan; 5 Department of Sustainable Health Science, Center for Preventive Medical Sciences, Chiba University, 1-33 Yayoi-cho, Inage-ku, Chiba 263-8522, Chiba, Japan; midoriy@faculty.chiba-u.jp; 6 Department of Food Management, School of Food, Agricultural and Environmental Sciences, Miyagi University, 2-2-1 Hatadate, Taihaku-ku, Sendai 982-0215, Miyagi, Japan; nishikaw@myu.ac.jp; 7 Faculty of Beauty & Wellness, Professional University of Beauty & Wellness, 3-9-3 Ushikubo, Tsuzuki-ku, Yokohama 224-0012, Kanagawa, Japan; kyoichi.oshida@gmail.com

**Keywords:** one-carbon metabolism, unmetabolized folic acid, 5-methyltetrahydrofolate, betaine, homocysteine, S-adenosylmethionine, transsulfuration pathway, cysteine, pregnant women, cord blood

## Abstract

**Background/Objectives**: Folic acid (FA) intake impacts one-carbon metabolism (OCM), which is crucial for fetal development and epigenetic regulation. While FA supplementation is known to lower homocysteine levels, its comprehensive effects on OCM-related metabolites in maternal and cord blood remain unclear. This study aimed to investigate the association between FA use and serum OCM-related metabolite profiles in Japanese pregnant women. **Methods**: We analyzed 146 mother-infant pairs from the Chiba study of Mother and Child Health (C-MACH) cohort. Blood samples were collected in early pregnancy, late pregnancy, and at delivery (maternal and cord blood). FA use was assessed via self-administered questionnaires. Serum concentrations of 18 OCM-related metabolites, including 5-methyltetrahydrofolate (5-MTHF) and homocysteine, were measured using LC-MS/MS. **Results**: FA users exhibited significantly higher 5-MTHF and lower total homocysteine concentrations in maternal blood at all time points and in cord blood compared to non-users. Compared to non-users, FA users exhibited a lower serine/glycine ratio in early pregnancy, a higher betaine/DMG ratio in maternal blood at delivery, and higher S-adenosylmethionine and total cysteine concentrations in maternal blood during late pregnancy. In cord blood, unmetabolized folic acid concentrations did not differ significantly between FA users and non-users. Furthermore, the cord-to-maternal 5-MTHF ratio was significantly lower in FA users. **Conclusions**: Our findings suggest that FA use during pregnancy may contribute to the optimization of OCM in both the mother and fetus.

## 1. Introduction

**Figure 1:**
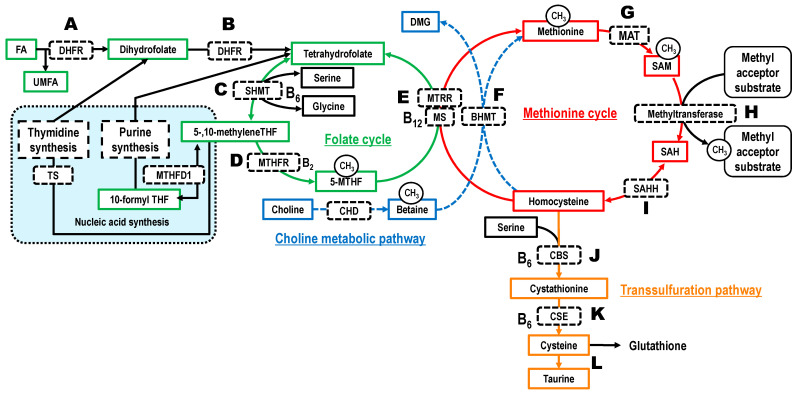
Overview of one-carbon metabolism. Abbreviations: 5-MTHF, 5-methyltetrahydrofolate; B_12_, cobalamin/methylcobalamin; B_2_, riboflavin; B_6_, pyridoxal phosphate (pyridoxine/pyridoxal/pyridoxamine); BAD, betaine aldehyde dehydrogenase; BHMT, betaine–homocysteine methyltransferase; CBS, cystationine-β synthase; CHD, choline dehydrogenase; CSE, cystathionine γ-lyase; DMG, dimethylglycine; UMFA, unmetabolized folic acid; MAT, methionine adenosyltransferase; MS, methionine synthase; MTHFR, methylenetetrahydrofolate reductase; MTRR, methionine synthase reductase; SAH, *S*-adenosylhomocysteine; SAHH, *S*-adenosylhomocysteine hydrolase; SAM, *S*-adenosylmethionine; SHMT, serine hydroxymethyltransferase; THF, tetrahydrofolate. Lines are color-coded to represent specific metabolic pathways: green for folate metabolism, blue for the choline metabolic pathway, red for the methionine cycle, and orange for the transsulfuration pathway. The alphabetical labels in the diagram correspond to the metabolic processes and enzymatic reactions discussed in main the text, A–D: The folate cycle, representing the metabolism of ingested folic acid (FA) to 5-methyltetrahydrofolate (5-MTHF), E–F: Remethylation of homocysteine to methionine by 5-MTHF (E) or betaine (F), G–I: The methionine cycle and S-adenosylmethionine (SAM)-dependent transmethylation reactions, J–L: The transsulfuration pathway, leading from homocysteine to the synthesis of cysteine and taurine. This metabolism diagram is reprinted from our previous article [14].

Extensive epidemiological studies have highlighted the concept of Developmental Origins of Health and Disease (DOHaD), suggesting that environmental factors during the fetal and early postnatal periods influence the risk of developing non-communicable diseases (NCDs). In the context of DOHaD, epigenetic modifications play a crucial role in gene expression and are considered underlying predispositions for NCDs in later life [1,2]. Epigenetic regulatory mechanisms include DNA methylation, histone modification, and non-coding RNA [3]. The fetal period involves particularly high epigenetic plasticity, making it highly susceptible to alterations induced by environmental factors [4]. Methyl groups required for DNA and histone methylation are supplied by *S*-adenosylmethionine (SAM), which is synthesized via one-carbon metabolism (OCM) [5]. Consequently, the demand for nutrients serving as methyl donors increases during pregnancy [6]. OCM is a metabolic network in which the folate cycle and choline metabolic pathway are linked to the methionine cycle, and homocysteine (Hcy) in the methionine cycle is further connected to the transsulfuration pathway (Figure 1). The primary role of OCM is to supply one-carbon units [7,8,9,10], which are essential intermediates for methyl transfer reactions [11] and biosynthetic processes such as nucleic acid synthesis [9,11]. Thus, understanding OCM during pregnancy, a period of dramatic epigenetic remodeling, is essential for DOHaD research. We previously reported that serum 5-methyltetrahydrofolate (5-MTHF) concentrations are associated with the metabolic dynamics of the choline metabolic pathway, the methionine cycle, and the transsulfuration pathway in pregnant and young women [12,13,14]. This suggests that folate status serves as a key regulator of OCM. We further considered it necessary to focus on exogenous environmental factors that affect folate status and to clarify these relationships comprehensively and longitudinally.

Various folate forms are involved in OCM. “Folic acid” (FA) refers to the synthetic pteroylmonoglutamic acid found in supplements and fortified foods, which is distinct from other folate molecular species [15]. In contrast, “dietary folate” refers to naturally occurring polyglutamate folate species found in foods, such as 5-MTHF and formyltetrahydrofolate [16,17,18]. FA is characterized by higher absorption in the small intestine compared to dietary folate [19,20]. Furthermore, as the most chemically stable form, FA is less susceptible to denaturation by heat, oxidation, specific pH conditions (pH 4–6) [21,22,23,24], or degradation by ultraviolet light [25]. Consequently, FA has been reported to exhibit higher bioavailability—defined as the proportion of ingested folate that is absorbed and available for metabolic processes [26]—compared to dietary folate. While the bioavailability of dietary folate is estimated at approximately 50% [15,19], the bioavailability of FA is estimated at 85% [18,22]. Therefore, the use of FA-containing supplements or FA-fortified foods facilitates meeting recommended folate intakes [22,27].

Given the high bioavailability of FA, we hypothesized that FA intake during pregnancy influences OCM (Figure 1). Numerous studies on pregnant women have reported that FA intake increases total blood folate concentrations and decreases total homocysteine (tHcy) levels [28]. However, while a few studies have examined the effects on specific folate species [such as FA (unmetabolized folic acid, UMFA) and 5-MTHF] [29,30,31,32], the choline metabolic pathway [33], or the methionine cycle [34,35], a comprehensive analysis of OCM-related metabolites remains lacking.

Therefore, this study aimed to investigate the association between the use of FA-containing products and serum OCM-related metabolite profiles in pregnant women.

## 2. Materials and Methods

### 2.1. Participants

This study is based on the “Chiba study of Mother and Child Health” (C-MACH), a cohort study conducted by the Center for Preventive Medical Sciences at Chiba University and the Research Institute for Science and Engineering at Waseda University. The purpose of C-MACH is to explore how genetic and environmental factors—specifically the intrauterine environment and postnatal living environment—affect child health and development [36]. Recruitment took place between February 2014 and June 2015. Pregnant women at less than 13 weeks of gestation visiting two hospitals in Chiba Prefecture (Yamaguchi Hospital, Onodera Ladies Clinic) and one in Saitama Prefecture (Aiwa Hospital) were informed about the study. Participants were then randomly selected from consenting candidates. Follow-up was discontinued in cases of miscarriage, stillbirth, withdrawal of consent, or hospital transfer. The present study included 146 mother–infant pairs recruited from Aiwa Hospital (Saitama) out of the 434 total participants in C-MACH [36]. Although an optional general nutrition education program was available, no specific nutritional guidance involving dietary assessment, such as monitoring supplement use or dietary intake, was provided.

### 2.2. Study Design

Blood samples were collected at four time points: early pregnancy (12 weeks), late pregnancy (32 weeks), and at delivery (maternal blood and umbilical cord venous blood). Serum OCM-related metabolites were analyzed from these samples. Concurrently with blood sampling in early and late pregnancy, self-administered questionnaires regarding lifestyle habits were distributed to assess the use of FA-containing products. Although this study is primarily a cross-sectional analysis examining the association between FA use and serum OCM metabolites in early and late pregnancy, it also includes longitudinal components regarding the relationship between FA use in late pregnancy and metabolite levels in maternal and cord blood at delivery. The study was conducted in accordance with the Declaration of Helsinki. The study protocol was approved by the Ethics Committees of the Chiba University Graduate School of Medicine (ID: 451, 8 November 2013; ID: 462, 4 December 2013; ID: 502, 28 May 2014), Waseda University (ID: 2013-G002 (3), 13 November 2015), and Kagawa Nutrition University (ID: 67, 6 July 2016).

### 2.3. Measurement of Serum OCM-Related Metabolites

Blood collection followed a standardized protocol. Hospital staff randomly collected blood into serum separator tubes; samples were centrifuged at 1700× *g* for 10 min within 2 h of collection. The supernatant (serum) was aliquoted (0.5 mL) and stored at −80 °C until analysis. Eighteen OCM-related metabolites [5-MTHF, UMFA (The presence of unmetabolized FA in the circulating blood indicates that the metabolic capacity of FA is exceeded), choline, betaine, dimethylglycine (DMG), homocysteic acid, methionine, SAM, SAH, total homocysteine (tHcy), cystathionine, total cysteine (tCys), taurine, serine, glycine, riboflavin, pyridoxamine, and pyridoxine] were quantified using isotope-dilution mass spectrometry (Serum homocysteic acid was undetectable in all samples and is therefore not reported in the results. Homocysteine and cysteine were measured as total amounts because their oxidized forms, linked by disulfide bonds, were converted to their reduced forms using a reducing agent.) [13,14]. Samples were processed under light-shielding conditions using consumables such as light-shielding vials. Calibration curves were measured every 24 h, and quality control serum was analyzed every 12 h. Measurements were performed in duplicate, and the mean value was used. Samples with undetectable peaks or a signal-to-noise ratio of less than 10 were defined as below the limit of quantification and assigned a concentration of 0.

### 2.4. Dietary Assessment and Definition of Folic Acid Users

Daily FA intake (µg/day) was calculated based on information from self-administered questionnaires (brand name, manufacturer, period of use, frequency, and dose) collected in early and late pregnancy, referencing methods from a previous study [37]. The estimated intake represents the average daily intake over the 4-week period preceding blood sampling. We identified the nutritional content per dose based on the brand and manufacturer information and multiplied this by the number of units consumed per dose. This value was then adjusted by a frequency coefficient based on reported usage. Coefficients were applied as follows: “Twice or more daily” was adjusted to the manufacturer’s maximum recommended daily dose; “Once daily” was multiplied by 1; “4–6 times/week” by 5/7; “2–3 times/week” by 2.5/7; and “Less than once/week” was estimated as 0.1. Missing or unclear responses regarding amount or frequency were imputed using the mode of the study population. Use was assumed to be continuous unless a cessation date was recorded. Participants who did not consume FA supplements or fortified foods within 4 weeks of blood sampling were assigned an intake of 0 µg/day. Participants with FA intake > 0 µg/day were defined as the FA user group, and those with 0 µg/day as the FA non-user group.

### 2.5. Dietary Folate Intake Assessment

Dietary folate intake (µg/day) was estimated using the brief-type self-administered diet history questionnaire (BDHQ) in early and late pregnancy and was energy-adjusted using the residual method. The BDHQ is a validated fixed-portion questionnaire that assesses the frequency of consumption of 58 food and beverage items over the past month [38]. Validation studies showed that energy-adjusted folate intake calculated from the BDHQ was, on average, 29 µg/day (8.3%) higher than that derived from 16-day semi-weighed dietary records, with a Pearson correlation coefficient of 0.52 [38]. To account for reporting errors, participants were defined as under-reporters if their energy intake was <0.5 times the Estimated Energy Requirement (EER) for Physical Activity Level I, and as over-reporters if >1.5 times the EER for Physical Activity Level III, based on the Dietary Reference Intakes for Japanese (2015) [39,40] (Table S1).

### 2.6. Maternal and Neonatal Information

Information on maternal age, alcohol and smoking habits in late pregnancy, passive smoking exposure, self-reported height, pre-pregnancy weight, household annual income, and maternal education level was obtained from questionnaires in early pregnancy, late pregnancy, and at 10 months postpartum. Pre-pregnancy body mass index (BMI) was calculated as weight (kg) divided by the square of height (m). Information on maternal age at delivery, marital status, parity, mode of delivery, gestational age, infant sex, birth weight, and birth length was obtained from medical records.

### 2.7. Statistical Analysis

Continuous variables with a normal distribution are presented as mean and standard deviation (SD), while those with a non-normal distribution are presented as median and interquartile range. Categorical data are presented as numbers and percentages (%). Participants were stratified by FA use. Differences between groups (users vs. non-users) were analyzed using Welch’s *t*-test for normally distributed variables, the Mann–Whitney U test for non-normally distributed variables, and Pearson’s chi-square test for categorical data. As most OCM-related metabolite concentrations were non-normally distributed, non-parametric tests were used for all metabolite comparisons. Correlations were assessed using Spearman’s rank correlation coefficient. Stratification for early pregnancy analysis used FA use data from the early pregnancy questionnaire. For analyses of late pregnancy maternal blood, delivery maternal blood, and cord blood, stratification was based on FA use data from the late pregnancy questionnaire. Differences in serum OCM-related metabolite concentrations among the four categories (0 μg/day, >0 to <400 μg/day, 400 μg/day, >400 μg/day) of FA intake were evaluated using Steel-Dwass tests, which were performed for pairwise comparisons. A *p*-value < 0.05 (two-tailed) was considered statistically significant. Statistical analyses were performed using JMP^®^ Student Edition (version 18.2.2., SAS Institute Inc., Cary, NC, USA).

## 3. Results

### 3.1. Characteristics of Study Participants

**Figure 2:**
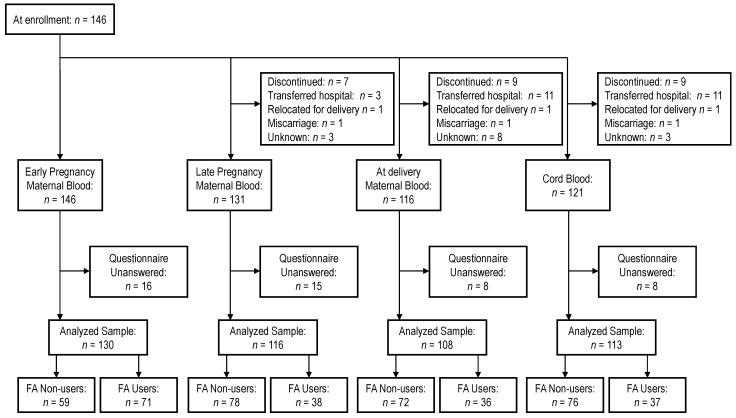
Flowchart of study participants. “Transferred hospital” refers to satogaeri shussan (returning to one’s hometown for delivery).

The final study population is shown in Figure 2. The number of participants with complete data on serum OCM concentrations and FA use was 130 in early pregnancy, 116 in late pregnancy, 108 at delivery, and 113 for cord blood. Table 1 presents the characteristics of the study participants, stratified by FA use (users vs. non-users) in early and late pregnancy. The proportion of FA users was 54.6% (71/130) in early pregnancy and 32.5% (38/116) in late pregnancy. Parity was significantly higher in FA non-users compared to FA users in early pregnancy, with a similar trend observed in late pregnancy. No significant differences were observed in neonatal characteristics between FA non-users and FA users.

### 3.2. Folic Acid and Dietary Folate Intake

Table 2 shows the distribution of FA intake. The distribution was uneven in both early and late pregnancy, with the 400 to <500 µg/day range being the most frequent. Table 3 presents the dietary folate intake in early and late pregnancy, stratified by FA use. BDHQ responses were missing for 16 participants in early pregnancy (8 non-users, 8 users) and 4 in late pregnancy (2 non-users, 2 users). Under-reporting of energy intake was identified in 12 participants in early pregnancy (8 non-users, 4 users) and 16 in late pregnancy (11 non-users, 5 users); no over-reporting was observed. There were no significant differences in energy-adjusted dietary folate intake derived from the BDHQ between FA users and non-users at either time point. This result remained non-significant even after excluding under-reporters.

### 3.3. Serum OCM-Related Metabolite Concentrations

Table 4 compares serum OCM-related metabolite concentrations in maternal blood (early pregnancy, late pregnancy, delivery) and cord blood, stratified by FA use.

#### 3.3.1. Folate Cycle

Serum 5-MTHF concentrations were significantly higher in the FA user group than in the non-user group across all maternal and cord blood samples. Serum UMFA concentrations were significantly higher in the FA user group only in early pregnancy. Furthermore, the serine/glycine ratio (precursor/product ratio of the serine hydroxymethyltransferase (SHMT) enzymatic reaction in the folate cycle) was significantly lower in the FA user group in early pregnancy, with a similar trend observed in late pregnancy (*p* = 0.056).

#### 3.3.2. Choline Metabolic Pathway

Regarding the betaine–homocysteine methyltransferase (BHMT) reaction (where betaine donates a methyl group to Hcy to form DMG), the FA user group exhibited significantly higher serum betaine concentrations in late pregnancy and a significantly higher betaine/DMG ratio at delivery compared to non-users. In cord blood, DMG concentrations were significantly lower, and the betaine/DMG ratio showed an increasing trend (*p* = 0.055) in the FA user group. Conversely, no significant differences were observed in serum choline concentrations in maternal or cord blood at any time point.

#### 3.3.3. Methionine Cycle

Regarding methionine cycle metabolites, serum methionine concentrations in cord blood were significantly higher in the FA user group compared to the non-user group. Serum SAM concentrations were significantly higher in the FA user group in late pregnancy, with similar trends observed in early pregnancy (*p* = 0.050) and cord blood (*p* = 0.060). No significant differences were found for SAH (a metabolite of SAM) or the SAM/SAH ratio. Serum tHcy concentrations were significantly lower in the FA user group than in the non-user group across all maternal and cord blood samples. Comparing medians, the differences (FA users vs. non-users) were −0.55 µmol/L (−9.6%) in early pregnancy, −0.84 µmol/L (−14.6%) in late pregnancy, −1.55 µmol/L (−20.8%) at delivery, and −1.01 µmol/L (−16.1%) in cord blood.

#### 3.3.4. Transsulfuration Pathway

In the transsulfuration pathway, serum tCys concentrations were significantly higher in the FA user group in late pregnancy, with a higher trend observed in cord blood (*p* = 0.066). The tHcy/tCys ratio was significantly lower in the FA user group compared to the non-user group across all maternal and cord blood samples. Notably, the statistical significance of the difference between groups was more pronounced when using the tHcy/tCys ratio (an indicator of enzymatic activity) compared to tHcy alone, as evidenced by lower *p*-values [tHcy: *p* = 8.01 × 10^−3^, 4.06 × 10^−3^, 7.73 × 10^−4^, 4.20 × 10^−4^ vs. tHcy/tCys ratio: *p* = 9.55 × 10^−4^, 1.40 × 10^−5^, 3.38 × 10^−6^, 3.14 × 10^−5^ for early pregnancy, late pregnancy, delivery, and cord blood, respectively].

#### 3.3.5. Vitamins

Serum riboflavin concentrations were significantly higher in the FA user group than in the non-user group across all maternal and cord blood samples. Pyridoxamine concentrations were significantly higher in the FA user group only in cord blood.

### 3.4. Correlations Between Maternal Blood at Delivery and Cord Blood

Table 5 presents the correlations of OCM-related metabolite concentrations between maternal blood at delivery and cord blood, stratified by FA use. Significant positive correlations were observed between maternal and cord blood for all metabolites, except for serum UMFA in the FA user group and pyridoxine in both groups.

### 3.5. Cord Blood to Maternal Blood Ratios

Table 6 shows the ratio of cord blood to maternal blood at delivery, stratified by FA use. Significant differences between the two groups were observed for 5-MTHF and cystathionine. The cord/maternal ratio for 5-MTHF was significantly higher in the FA non-user group compared to the user group, whereas the ratio for cystathionine was significantly lower in the non-user group.

## 4. Discussion

This study investigated the association between the use of FA-containing products and serum OCM-related metabolites in Japanese pregnant women. We found that FA users exhibited significantly higher serum 5-MTHF and riboflavin concentrations, and significantly lower tHcy concentrations and tHcy/tCys ratios in both maternal and cord blood throughout pregnancy compared to non-users. Furthermore, FA use reduced the 5-MTHF ratio in cord blood relative to maternal blood without causing UMFA accumulation in cord blood.

As previously reported, the participants in this study had a relatively high socioeconomic status [12]. A preceding study of Japanese pregnant women reported that FA supplement use was independently associated with age, marital status, education level, family income, BMI, and parity, among other factors [41]. It is well established that primiparous women are more likely to use FA supplements than multiparous women [42]. In our study, only parity was associated with FA use in early pregnancy. Therefore, it should be noted that our study population had a higher FA usage rate than the representative Japanese population and that the FA user group included more primiparous women.

Serum 5-MTHF concentrations in maternal and cord blood in this study were compared with representative values from previous studies (Table S2). Maternal blood concentrations in the FA non-user group were comparable to those in populations from countries without mandatory FA fortification [31,43]. Conversely, maternal blood concentrations in the FA user group were comparable to, and cord blood concentrations were slightly higher than, those in populations from countries with mandatory FA fortification [29,44,45,46,47,48]. Serum UMFA concentrations in both maternal and cord blood, even among FA users, were similar to representative values from countries without mandatory fortification [31,49] and lower than most values from countries with mandatory fortification [29,30,44,45,47,50,51,52,53] (Table S2). This suggests that while our participants consumed more FA than typical Japanese cohorts, the impact of UMFA accumulation was minimal.

In this study, the FA user group showed significantly higher 5-MTHF levels in maternal blood (early, late, delivery) and cord blood compared to non-users. Previous studies also found that in intervention trials involving pregnant women, maternal blood 5-MTHF concentrations increased following supplementation with 400 µg of folic acid from 14 to 36 weeks of gestation, consistent with the results of this study [29]. Consistent with studies tracing isotope-labeled FA [54,55], our results suggest that ingested FA is absorbed in the small intestine, metabolized primarily in the liver via the folate cycle, and converted to 5-MTHF (Figure 1A–D). Furthermore, the serine/glycine ratio was significantly lower in the FA user group in early pregnancy, with a similar downward trend in late pregnancy. A previous study involving 12 healthy men and women also demonstrated that the blood serine/glycine ratio decreased after FA intervention (5 mg for 3 weeks) compared to before intervention, showing similar results despite differences in subject characteristics and FA intake levels [34]. This implies that in the SHMT reaction—part of the pathway metabolizing FA to 5-MTHF—the flux is shifted towards serine (precursor) consumption and glycine (product) production more actively in FA users (Figure 1C). In silico mathematical modeling has also reported that serine concentrations rise when folate status is low [56]. In this study, a positive correlation was found between maternal and cord blood (Table 5). The cord blood/maternal blood 5-MTHF ratio was significantly lower in the FA user group (Table 6). This suggests that while umbilical cord blood 5-MTHF depends on maternal concentration, when fetal 5-MTHF levels are low, more 5-MTHF may be transported from the mother to the fetus via the placenta to maintain fetal 5-MTHF levels (Figure S1).

In this study, serum UMFA concentrations in early pregnancy were significantly higher in the FA user group. However, consistent with previous findings [30,31], cord blood UMFA concentrations did not differ significantly by FA use. As discussed in our previous report [12], this suggests that FA does not accumulate in cord blood, at least at delivery, in our population. Furthermore, as shown in Table 5, a significant positive correlation between maternal and cord blood UMFA concentrations was observed in the FA non-user group, whereas no significant correlation was found in the FA user group. This suggests that when cord blood 5-MTHF concentrations are sufficient, the transfer of FA from maternal to cord blood may decrease. Notably, the presence of detectable UMFA in the blood of non-users is consistent with previous reports [31]; in this study, it implies that the FA non-user group may have had unreported exposure to FA-fortified products not captured by the questionnaire.

Serum tHcy concentrations were significantly lower in the FA user group. This is likely due to increased remethylation of Hcy to methionine by methionine synthase (Figure 1E) and increased catabolism via the transsulfuration pathway [13] (Figure 1J,K). Although FA supplementation is not typically emphasized in late pregnancy, it reduced tHcy by 1.55 µmol/L (−20.8%) in maternal blood at delivery and 1.01 µmol/L (−16.1%) in cord blood (Figure S2). The clinical benefits of this reduction warrant further investigation.

Regarding the choline metabolic pathway, the FA user group exhibited higher betaine (precursor) and a trend toward lower DMG (product) compared to non-users (Figure 1F). In a previous observational study of Spanish pregnant women prescribed 400 µg/day of FA until early pregnancy, the group that discontinued FA use after early pregnancy exhibited significantly lower blood betaine concentrations compared to the group that continued use [33]. Additionally, studies have shown that when blood folate concentrations are dichotomized, the lower folate group exhibits higher blood DMG and lower blood betaine concentrations compared to the higher folate group [14,33]. Similarly, an intervention study involving 500 healthy adults reported that the rise in plasma tHcy concentrations following methionine loading was negatively correlated with plasma betaine concentrations, and this relationship was more pronounced in subjects with low blood folate concentrations [57]. These findings suggest a “betaine-sparing” effect: high 5-MTHF levels in FA users provide sufficient methyl groups for Hcy remethylation, reducing the need for the alternative betaine-dependent pathway, thereby conserving betaine and reducing DMG production (Figure 1E,F).

Serum SAM concentrations were significantly higher in the FA user group in late pregnancy, with a similar trend in early pregnancy (*p* = 0.050). A previous study that traced metabolism by intravenously administering labeled methionine to healthy women and pregnant women in early, mid, and late pregnancy reported that methionine transmethylation is enhanced in late pregnancy [58]. The significant difference observed only in late pregnancy suggests that 5-MTHF is efficiently utilized to increase the SAM supply in response to the demand for SAM-dependent transmethylation reactions (Figure 1H) during this period. If this increase in SAM concentrations is reflected in the fetus, it could influence fetal epigenetics. Previous studies have reported that FA intake is associated with DNA methylation in offspring [35,59,60,61], and our results may provide a mechanistic explanation for this relationship. Serum tHcy concentrations in maternal and cord blood were lower in the FA user group compared to the FA non-user group. These results are consistent with the 5-MTHF concentration-dependent relationship we previously reported [12]. Furthermore, the FA user group exhibited a lower tHcy/tCys ratio compared to the non-user group. As we previously reported, elevated 5-MTHF levels may inhibit glycine N-methyltransferase, suppressing methylation reactions and consequently increasing SAM [14]. This SAM may inhibit methylene tetrahydrofolate reductase, BHMT, and methionine adenosyltransferase, while activating CBS, thereby increasing flux into the transsulfuration pathway and consequently lowering homocysteine [14]. This may have implications for fetal growth. Since fetal hepatic cystathionine γ-lyase (Figure 1K) is inactive [62,63,64], cysteine cannot be synthesized in vivo and may be considered an essential amino acid for the fetus and neonate [63,65,66,67], meaning its supply relies on placental transport from the mother. Moreover, taurine, synthesized from cysteine, plays roles in fetal development and cytoprotection [68,69,70,71]. Future studies should investigate the possibility that the reduction in Hcy associated with FA use involves enhanced metabolism via the transsulfuration pathway.

This study has several limitations. First, OCM-related metabolites in blood do not necessarily directly reflect OCM dynamics within organ cells. Furthermore, while a positive correlation was observed between maternal and fetal OCM-related metabolite concentrations, it remains unclear whether these metabolites actually transferred from maternal blood to umbilical cord blood or were metabolized within the fetus. Second, the BDHQ used to estimate dietary folate is also self-reported, and underreporting is common among pregnant women [72,73]. We attempted to mitigate this by using energy-adjusted data [74]. Third, we could not calculate choline or betaine intake from the BDHQ, so dietary influence on choline metabolism was not considered. Fourth, the higher riboflavin and pyridoxamine levels in the FA user group suggest these vitamins may have been present in the supplements or fortified foods, acting as confounders. Fifth, single nucleotide polymorphisms may influence OCM [75]; however, they have not been considered in this study. Sixth, participants were not fasting at the time of blood collection, which may have influenced the results. Previous studies have reported differences between groups when blood UMFA concentrations and blood 5-MTHF concentrations were compared between participants who fasted for 8 h or more and those who did not [76]. Finally, the estimation of FA intake may be imprecise due to several factors. Primarily, the validity of the questionnaire used to assess FA usage has not been established. Regarding the reporting of FA use, certain FA-containing products, such as cereals and dairy beverages, may not clearly indicate FA fortification on their packaging, nor were they explicitly listed as examples in the questionnaire. Consequently, participants may have inadvertently omitted these sources. Additionally, although we adopted the calculation method for FA intake (µg/day) from a previous study, it remains questionable whether calculating intake based solely on use within the 4 weeks preceding blood sampling accurately reflects true intake. This is because intervention trials have reported differences in blood folate concentrations between weekly and daily FA intake [77], suggesting that continuity (frequency) is a critical factor, which was not considered in this study. Furthermore, although folate is a water-soluble vitamin, it is primarily stored in the liver as polyglutamates. A study involving young Chinese women reported that after a 9-month FA intervention (100–4000 µg/day), folate concentrations did not return to baseline even after 3 months [77]. Conversely, an intervention study in healthy men reported that continuous FA intake of 400 µg/day for 13 weeks is required to reach a plateau [78]. In this study, participants who had consumed FA but discontinued use more than 4 weeks prior were defined as having an intake of 0 µg/day, and the analysis included users who may have stopped or started within the 4-week window. These definitional inaccuracies may have introduced classification errors. Moreover, stratification for maternal blood at delivery and cord blood was based on data from late pregnancy, and it is unclear whether FA intake continued similarly thereafter. As no calculation method accounting for these temporal factors has been reported in previous studies, we prioritized data reliability by performing the primary analysis using a broad categorization of FA users versus non-users. However, as supplementary analyses, we evaluated the correlations between serum OCM-related metabolite concentrations and estimated FA intake (µg/day) as continuous variables (Table S3). Furthermore, we categorized the participants into four groups (0 µg/day, >0 to <400 µg/day, 400 µg/day, and >400 µg/day) based on the 400 µg/day threshold generally recommended for preventing neural tube defects, and performed pairwise inter-group comparisons for all serum OCM-related metabolite concentrations (Table S4). These analyses yielded results highly consistent with those presented in the main text. Interestingly, even a low-dose FA intake of less than 400 µg/day resulted in significantly higher 5-MTHF concentrations compared to the 0 µg/day group.

## 5. Conclusions

This study showed that the use of FA-containing supplements or fortified foods by Japanese pregnant women was associated with higher folate status (5-MTHF concentrations) and lower homocysteine concentrations in maternal blood during pregnancy and cord blood. Importantly, FA use was associated with alterations in one-carbon metabolism dynamics, suggesting a potential sparing of betaine, increased availability of SAM, and enhanced cysteine synthesis via the transsulfuration pathway. These metabolic associations were observed without the accumulation of UMFA in cord blood. Our findings suggest that FA use during pregnancy may contribute to optimizing methyl donor supply and amino acid availability for both the mother and the fetus, beyond the well-known prevention of neural tube defects in early pregnancy.

## Figures and Tables

**Table 1:** Characteristics of pregnant women and newborns stratified by folic acid use in early or late pregnancy.

			Early Pregnancy							Late Pregnancy						
			FA Non-Users (*n* = 59)			FA Users (*n* = 71)			*p*-Value ^b^	FA Non-Users (*n* = 78)			FA Users (*n* = 38)			*p*-Value ^b^
			*n*	% ^a^	Variable ^b^	*n*	% ^a^	Variable ^b^		*n*	% ^a^	Variable ^b^	*n*	% ^a^	Variable ^b^	
Maternal Characteristics																
	Maternal age at delivery (years)		59		31.8 (4.9)	71		32.6 (4.4)	0.288	78		32.1 (4.6)	38		32.4 (5.0)	0.743
		<20	0	0.0		1	1.4		0.418	0	0.0		1	2.6		0.753
		20 to <25	4	6.8		1	1.4			4	5.1		1	2.6		
		25 to <30	16	27.1		18	25.4			22	28.2		11	28.9		
		30 to <35	21	35.6		27	38.0			27	34.6		12	31.6		
		35 to <40	13	22.0		21	29.6			20	25.6		11	28.9		
		≥40	5	8.5		3	4.2			5	6.4		2	5.3		
	Pre-pregnancy BMI (kg/m^2^)		59		21.1 (19.5, 24.0)	71		20.8 (19.9, 23.5)	0.868	78		21.0 (19.5, 23.4)	38		20.8 (20.4, 23.9)	0.389
		<18.5	6	10.2		5	7.0		0.605	7	9.0		3	7.9		0.600
		18.5 to <25	43	72.9		57	80.3			62	79.5		28	73.7		
		≥25	10	16.9		9	12.7			9	11.5		7	18.4		
		Missing	0	-		0	-			0	-		0	-		
	Marital status															
		Married	58	98.3		71	100.0		0.271	77	98.7		38	100.0		0.483
		Unmarried	1	1.7		0	0.0			1	1.3		0	0.0		
		Divorced/widowed	0	0.0		0	0.0			0	0.0		0	0.0		
		Missing	0	0.0		0	0.0			0	0.0		0	0.0		
	Parity		55	1 (1, 1)		61	1 (0, 1)		0.027	74	1 (0, 1)		33	1 (0, 1)		0.052
		0	11	20.0		25	41.0		0.050	19	25.7		16	48.5		0.067
		1	31	56.4		26	42.6			40	54.1		12	36.4		
		≥2	13	23.6		10	16.4			15	20.3		5	15.2		
		Missing	4	-		10	-			4	-		5	-		
	Educational attainment (years)															
		9	1	2.2		1	1.7		0.467	2	3.0		0	0.0		0.646
		12	13	28.9		12	20.0			14	20.9		10	27.8		
		13 to <16	20	44.4		24	40.0			29	43.3		14	38.9		
		≥16	11	24.4		23	38.3			22	32.8		12	33.3		
		Missing	14	-		11	-			11	-		2	-		
	Annual household income (JPY)															
		<2 million	0	0.0		0	0.0		0.174	0	0.0		0	0.0		0.202
		2 to <4 million	12	24.0		10	15.6			17	22.7		5	13.9		
		4 to <6 million	15	30.0		17	26.6			25	33.3		8	22.2		
		6 to <8 million	13	26.0		19	29.7			19	25.3		12	33.3		
		8 to <10 million	4	8.0		11	17.2			7	9.3		6	16.7		
		≥10 million	3	6.0		7	10.9			4	5.3		5	13.9		
		I don’t know	3	6.0		0	0.0			3	4.0		0	0.0		
		Missing	9	-		7	-			3	-		2	-		
	Alcohol consumption ^c^															
		Never drank	52	100.0		63	95.5		0.297	76	98.7		36	94.7		0.312
		Drinkers during pregnancy	0	0.0		3	4.5			1	1.3		2	5.3		
		Missing	7	-		5	-			1	-		0	-		
	Smoking habits ^c^															
		Never smoked	47	90.4		55	77.5		0.982	71	91.0		35	92.1		0.182
		Ex-smokers who quit before pregnancy	4	7.7		14	19.7			6	7.7		2	5.3		
		Smokers during pregnancy	1	1.9		2	2.8			1	1.3		1	2.6		
		Missing	7	-		0	-			0	-		0	-		
	Passive smoking								0.183							0.364
		No	40	67.8		57	80.3			62	79.5		32	84.2		
		Yes	12	20.3		9	12.7			16	20.5		5	13.2		
		Missing	7	11.9		5	7.0			0	0.0		1	2.6		
Neonatal Characteristics																
	Sex															
		Male	27	52.9		28	43.1		0.291	40	53.3		16	42.1		0.259
		Female	24	47.1		37	56.9			35	46.7		22	57.9		
		Missing	8	-		6	-			3	-		0	-		
	Birth weight (g)		51		3121(400)	65		3177 (366)	0.440	75		3165 (401)	38		3100 (295)	0.332
		<2500	3	5.9		2	3.1		0.716	4	5.3		1	2.6		0.471
		2500–3999	47	92.2		60	93.8			69	92.0		37	97.4		
		≤4000	1	2.0		2	3.1			2	2.7		0	0.0		
		Missing	8	-		7	-			3	-		0	-		
	Birth length (cm)		51		49.6 (2.0)	65		49.8 (1.7)	0.504	75		49.7 (1.9)	38		49.7 (1.6)	0.988
	Gestational age at birth (weeks)		51		39.4 (38.7, 40.4)	65		39.7 (38.6, 40.3)	0.938	75		39.6 (38.7, 40.4)	38		39.3 (38.4, 40.2)	0.189
		Preterm births (<37 weeks)	2	3.9		0	0.0		0.107	2	2.7		0	0.0		0.310
		Term births (37 to <42 weeks)	49	96.1		65	100.0			73	97.3		38	100.0		
		Postterm births (≥42 weeks)	0	0.0		0	0.0			0	0.0		0	0.0		
		Missing	8	-		6	-			3	-		0	-		
	Type of delivery															
		Vaginal	41	87.2		45	86.5		0.919	61	91.0		24	80.0		0.127
		Caesarean	6	12.8		7	13.5			6	9.0		6	20.0		
		Missing	12	-		19	-			11	-		8	-		

Individuals with an FA intake of 0 µg/day within the past 4 weeks were classified as “FA non-users”; those with >0 µg/day were classified as “FA users”. ^a^ Percentage of valid responses in each group; ^b^ Continuous variables following a normal distribution were expressed as mean (standard deviation), and comparisons between two groups were performed using Welch’s *t*-test. Continuous variables not following a normal distribution were expressed as median (25th, 75th percentile), and comparisons between two groups were performed using the Mann–Whitney U test. A Pearson chi-square test was conducted to investigate the relationship between the categories of FA non-use and FA use.; ^c^ Obtained from the questionnaire for the late pregnancy period.

**Table 2:** Distribution of FA intake in early and late pregnancy.

	Early Pregnancy		Late Pregnancy	
FA Intake, µg/Day ^a^	*n*	%	*n*	%
0	59	45.4	78	67.2
>0 to <100	6	4.6	3	2.6
100 to <200	3	2.3	6	5.2
200 to <300	14	10.8	5	4.3
300 to <400	7	5.4	0	0.0
400 to <500	32	24.6	22	19.0
500 to <600	3	2.3	2	1.7
≥600	6	4.6	0	0.0

^a^ Daily folic acid intake calculated from records of supplement and fortified food use.

**Table 3:** Food-derived folate intake in early and late pregnancy stratified by use of folic acid ^a,b^.

		Early Pregnancy (n = 130)							Late Pregnancy (n = 116)						
		FA Non-Users (n = 59)			FA Users (n = 71)				FA Non-Users (n = 78)			FA Users (n = 38)			
		*n*	Median	(25th, 75th)	*n*	Median	(25th, 75th)	*p*-Value ^c^	*n*	Median	(25th, 75th)	*n*	Median	(25th, 75th)	*p*-Value ^c^
Energy-adjusted dietary folate intake ^d^ (µg)															
	Overall ^e^	51	204	(237, 163)	63	205	(253, 169)	0.482	76	228	(269, 180)	36	244	(292, 187)	0.328
	Exclude underreporters ^f^	43	217	(256, 179)	59	218	(262, 180)	0.858	65	239	(293, 190)	31	251	(305, 196)	0.413
	BDHQ Unanswered	8	-	-	8	-	-	-	2	-	-	2	-	-	-

Continuous variables are expressed as median (25th, 75th percentile). ^a^ Individuals with an FA intake of 0 µg/day or less within the past 4 weeks were classified as “FA non-users,” while those with an intake of >0 µg/day were classified as “FA users.”; ^b^ Estimated daily dietary folate intake (µg/day) from BDHQ; ^c^ *p*-value from the Mann–Whitney U test comparing dietary folate intake between the FA non-user group and the FA user group.; ^d^ Using the residual method for energy adjustment of folate intake; ^e^ Values excluding 8 non-FA users and 8 FA users in early pregnancy and 2 non-FA users and 2 FA users in late pregnancy who did not respond to BDHQ. ^f^ Values after excluding 8 non-FA users and 4 FA users in early pregnancy, and 11 non-FA users and 5 FA users in late pregnancy. Individuals underreporting their dietary intake were defined as those whose estimated energy intake deficiency (kcal/day) was less than half of the estimated energy requirement for women aged 18–29 years at activity level I, based on the 2015 Dietary Reference Intakes.

**Table 4:** Comparison of serum OCM-related metabolite concentrations in maternal blood and umbilical cord blood during early pregnancy, late pregnancy, and delivery, stratified by FA use ^a^.

			FA Non-Users		FA Users		*p*-Value ^b^
	Analytes and Metabolic Indicators	Samples	*n*	Median (25th, 75th)	*n*	Median (25th, 75th)	
Folate Cycle	5-MTHF, nmol/L	Maternal blood in early pregnancy	59	19.8 (16.2, 27.5)	71	47.2 (35.0, 58.8)	<0.0001
		Maternal blood in late pregnancy	78	13.7 (10.8, 19.2)	38	47.9 (25.7, 62.7)	<0.0001
		Maternal blood at delivery	72	11.2 (9.4, 16.2)	36	35.5 (17.8, 48.7)	<0.0001
		Cord blood	76	41.0 (33.1, 51.6)	37	66.0 (51.0, 93.1)	<0.0001
	UMFA, nmol/L	Maternal blood in early pregnancy	59	0.545 (0.000, 0.910)	71	0.715 (0.145, 1.706)	0.033
		Maternal blood in late pregnancy	78	0.599 (0.000, 1.195)	38	0.792 (0.364, 1.249)	0.104
		Maternal blood at delivery	72	0.434 (0.000, 0.799)	36	0.450 (0.000, 1.521)	0.407
		Cord blood	76	0.530 (0.139, 1.098)	37	0.543 (0.000, 1.016)	0.961
Choline Metabolic Pathway	Choline, µmol/L	Maternal blood in early pregnancy	59	6.94 (6.05, 8.84)	71	7.60 (6.64, 9.06)	0.149
		Maternal blood in late pregnancy	78	7.78 (6.88, 9.73)	38	8.90 (6.78, 10.06)	0.582
		Maternal blood at delivery	72	11.58 (9.96, 12.76)	36	11.10 (9.10, 13.51)	0.789
		Cord blood	76	28.70 (25.15, 31.79)	37	27.90 (23.93, 33.58)	0.833
	Betaine, µmol/L	Maternal blood in early pregnancy	59	21.4 (17.0, 25.5)	71	21.2 (17.3, 25.5)	0.870
		Maternal blood in late pregnancy	78	13.1 (11.7, 15.2)	38	14.9 (11.8, 18.4)	0.021
		Maternal blood at delivery	72	13.5 (11.8, 15.7)	36	15.2 (11.6, 17.4)	0.181
		Cord blood	76	26.4 (23.4, 31.5)	37	27.6 (25.0, 31.0)	0.373
	DMG, µmol/L	Maternal blood in early pregnancy	59	1.83 (1.24, 2.54)	71	1.72 (1.17, 2.43)	0.583
		Maternal blood in late pregnancy	78	1.57 (1.09, 2.45)	38	1.71 (1.03, 2.17)	0.674
		Maternal blood at delivery	72	2.38 (1.72, 3.75)	36	1.85 (1.51, 2.75)	0.096
		Cord blood	76	3.33 (2.68, 4.21)	37	2.89 (2.10, 3.56)	0.037
	Betaine/DMG	Maternal blood in early pregnancy	59	11.5 (9.5, 16.1)	71	12.5 (9.1, 17.4)	0.486
		Maternal blood in late pregnancy	78	8.4 (6.0, 11.4)	38	8.5 (6.6, 15.3)	0.213
		Maternal blood at delivery	72	5.6 (3.9, 8.1)	36	7.1 (5.2, 9.1)	0.011
		Cord blood	76	8.3 (6.3, 10.7)	37	10.2 (6.9, 13.6)	0.055
Methionine Cycle	Methionine, µmol/L	Maternal blood in early pregnancy	59	18.7 (16.4, 22.8)	71	19.7 (16.5, 25.8)	0.287
		Maternal blood in late pregnancy	78	19.0 (16.6, 22.9)	38	19.6 (17.0, 22.6)	0.410
		Maternal blood at delivery	72	20.2 (17.8, 23.4)	36	20.8 (18.1, 24.6)	0.623
		Cord blood	76	29.4 (27.3, 32.2)	37	30.4 (28.9, 36.0)	0.040
	SAM, nmol/L	Maternal blood in early pregnancy	59	56.1 (48.6, 64.2)	71	59.8 (53.5, 71.6)	0.050
		Maternal blood in late pregnancy	78	56.3 (48.9, 63.6)	38	62.3 (52.5, 70.5)	0.019
		Maternal blood at delivery	72	58.3 (49.6, 66.9)	36	63.2 (51.1, 71.7)	0.186
		Cord blood	76	112.0 (96.7, 128.3)	37	119.5 (106.0, 138.8)	0.060
	SAH, nmol/L	Maternal blood in early pregnancy	59	11.1 (9.0, 13.9)	71	11.1 (9.5, 13.4)	0.654
		Maternal blood in late pregnancy	78	12.4 (9.8, 14.0)	38	12.5 (9.7, 14.7)	0.709
		Maternal blood at delivery	72	22.1 (18.6, 28.4)	36	25.9 (19.7, 33.7)	0.158
		Cord blood	76	42.4 (37.3, 50.4)	37	46.1 (38.1, 62.9)	0.132
	SAM/SAH	Maternal blood in early pregnancy	59	5.35 (3.97, 6.25)	71	5.41 (4.33, 6.42)	0.449
		Maternal blood in late pregnancy	78	4.76 (3.87, 5.61)	38	4.78 (4.31, 6.48)	0.319
		Maternal blood at delivery	72	2.68 (1.90, 3.41)	36	2.59 (1.79, 3.13)	0.747
		Cord blood	76	2.63 (2.09, 3.25)	37	2.52 (1.76, 3.10)	0.567
Transulfuration Pathway	tHcy, µmol/L	Maternal blood in early pregnancy	59	5.73 (4.87, 6.49)	71	5.18 (4.44, 5.83)	0.008
		Maternal blood in late pregnancy	78	5.76 (4.93, 7.34)	38	4.92 (4.16, 6.33)	0.004
		Maternal blood at delivery	72	7.46 (6.50, 9.30)	36	5.91 (4.85, 7.69)	0.001
		Cord blood	76	6.29 (5.42, 7.89)	37	5.28 (4.06, 6.55)	0.0004
	Cystathionine, nmol/L	Maternal blood in early pregnancy	59	105 (82, 133)	71	100 (73, 130)	0.682
		Maternal blood in late pregnancy	78	218 (178, 288)	38	195 (162, 272)	0.373
		Maternal blood at delivery	72	219 (183, 315)	36	198 (143, 261)	0.044
		Cord blood	76	324 (238, 405)	37	318 (259, 358)	0.764
	tCys, µmol/L	Maternal blood in early pregnancy	59	233 (214, 258)	71	242 (219, 262)	0.229
		Maternal blood in late pregnancy	78	210 (198, 222)	38	221 (207, 239)	0.008
		Maternal blood at delivery	72	232 (216, 262)	36	250 (219, 263)	0.181
		Cord blood	76	211 (195, 225)	37	218 (200, 238)	0.066
	tHcy/tCys × 10^2^	Maternal blood in early pregnancy	59	2.41 (2.14, 2.88)	71	2.14 (1.8, 2.38)	0.0002
		Maternal blood in late pregnancy	78	2.79 (2.51, 3.38)	38	2.18 (1.94, 2.73)	< 0.0001
		Maternal blood at delivery	72	3.10 (2.82, 3.68)	36	2.36 (2.13, 3.01)	< 0.0001
		Cord blood	76	3.09 (2.7, 3.56)	37	2.35 (1.83, 2.99)	< 0.0001
	Taurine, µmol/L	Maternal blood in early pregnancy	59	64.1 (52.2, 90.0)	71	79.3 (55.5, 103.0)	0.132
		Maternal blood in late pregnancy	78	61.1 (49.9, 78.8)	38	55.6 (48.7, 77.3)	0.420
		Maternal blood at delivery	72	72.0 (49.7, 105.3)	36	76.6 (55.0, 103.2)	0.720
		Cord blood	76	183.3 (148.9, 226.1)	37	185.5 (139.0, 235.0)	0.785
Amino Acid	Serine, µmol/L	Maternal blood in early pregnancy	59	100 (89, 113)	71	96 (86, 107)	0.076
		Maternal blood in late pregnancy	78	104 (91, 117)	38	103 (91, 110)	0.459
		Maternal blood at delivery	72	116 (101, 130)	36	110 (98, 128)	0.434
		Cord blood	76	153 (142, 164)	37	158 (138, 172)	0.653
	Glycine, µmol/L	Maternal blood in early pregnancy	59	152 (131, 167)	71	154 (141, 183)	0.202
		Maternal blood in late pregnancy	78	145 (127, 171)	38	154 (136, 175)	0.296
		Maternal blood at delivery	72	172 (139, 205)	36	179 (148, 208)	0.390
		Cord blood	76	256 (235, 275)	37	264 (238, 285)	0.373
	Serine/Glycine	Maternal blood in early pregnancy	59	0.681 (0.554, 0.756)	71	0.605 (0.531, 0.680)	0.001
		Maternal blood in late pregnancy	78	0.701 (0.602, 0.806)	38	0.661 (0.601, 0.732)	0.056
		Maternal blood at delivery	72	0.663 (0.580, 0.758)	36	0.622 (0.554, 0.732)	0.250
		Cord blood	76	0.605 (0.554, 0.654)	37	0.604 (0.540, 0.627)	0.535
Co-factor	Riboflavin, nmol/L	Maternal blood in early pregnancy	59	7.84 (0.00, 14.30)	71	13.69 (5.05, 25.45)	0.007
		Maternal blood in late pregnancy	78	5.59 (0.00, 12.53)	38	13.07 (3.97, 24.21)	0.011
		Maternal blood at delivery	72	4.95 (0.00, 12.34)	36	10.06 (7.10, 21.04)	0.003
		Cord blood	76	50.85 (32.00, 69.58)	37	60.60 (37.03, 102.68)	0.039
	Pyridoxamine, nmol/L	Maternal blood in early pregnancy	59	0.218 (0.168, 0.276)	71	0.223 (0.175, 0.271)	0.768
		Maternal blood in late pregnancy	78	0.203 (0.174, 0.267)	38	0.236 (0.188, 0.282)	0.146
		Maternal blood at delivery	72	0.228 (0.187, 0.302)	36	0.253 (0.213, 0.306)	0.109
		Cord blood	76	0.287 (0.251, 0.409)	37	0.371 (0.299, 0.465)	0.004
	Pyridoxine, nmol/L	Maternal blood in early pregnancy	59	0.108 (0.070, 0.180)	71	0.138 (0.097, 0.212)	0.052
		Maternal blood in late pregnancy	78	0.121 (0.066, 0.191)	38	0.148 (0.092, 0.186)	0.189
		Maternal blood at delivery	72	0.122 (0.071, 0.166)	36	0.132 (0.064, 0.171)	0.679
		Cord blood	76	0.191 (0.117, 0.302)	37	0.242 (0.150, 0.431)	0.137

Variables are expressed as median (25th, 75th percentile). ^a^ Maternal blood at delivery and cord blood were stratified based on late pregnancy criteria; ^b^ The difference in serum concentrations of OCM-related metabolites between the FA non-users and the FA users was tested using the Mann–Whitney U test.

**Table 5:** Correlation between maternal blood at birth and cord blood concentrations of OCM-related metabolites stratified by FA use during delivery.

	FA Non-Users (*n* = 78)		FA Users (*n* = 38)	
Analytes	ρ	*p*-Value	ρ	*p*-Value
5-MTHF	0.406	0.0004	0.821	<0.0001
UMFA	0.459	<0.0001	0.244	0.157
Choline	0.351	0.003	0.579	0.0003
Betaine	0.335	0.004	0.417	0.013
DMG	0.808	<0.0001	0.811	<0.0001
Methionine	0.449	<0.0001	0.428	0.010
SAM	0.449	<0.0001	0.334	0.050
SAH	0.316	0.007	0.453	0.006
tHcy	0.771	<0.0001	0.770	<0.0001
Cystathionine	0.622	<0.0001	0.486	0.003
tCys	0.684	<0.0001	0.352	0.038
Taurine	0.054	0.651	0.301	0.079
Serine	0.269	0.022	0.508	0.002
Glycine	0.548	<0.0001	0.559	0.0005
Riboflavin	0.607	<0.0001	0.707	<0.0001
Pyridoxamine	0.227	0.056	0.279	0.105
Pyridoxine	0.422	0.0002	0.379	0.025

ρ: Spearman correlation coefficient.

**Table 6:** Cord blood/maternal blood at birth ratio stratified by FA use.

	FA Non-Users (*n* = 78)		FA Users (*n* = 38)		
	Median	(25th, 75th)	Median	(25th, 75th)	*p*-Value ^a^
5-MTHF	3.65	(2.78, 4.56)	2.12	(1.63, 2.77)	<0.0001
UMFA	1.02	(0.47, 1.46)	0.64	(0.18, 2.16)	0.559
Coline	2.48	(2.18, 3.06)	2.58	(2.14, 2.95)	0.819
Betaine	1.98	(1.70, 2.28)	1.90	(1.56, 2.18)	0.179
DMG	1.28	(1.15, 1.68)	1.46	(1.06, 1.89)	0.403
Methionine	1.44	(1.32, 1.66)	1.49	(1.32, 1.75)	0.511
SAM	1.94	(1.71, 2.30)	1.89	(1.66, 2.16)	0.570
SAH	1.87	(1.57, 2.25)	1.84	(1.60, 2.34)	0.666
tHcy	0.87	(0.77, 0.95)	0.84	(0.73, 0.90)	0.273
Cystathionine	1.31	(1.07, 1.58)	1.52	(1.31, 2.02)	0.023
tCys	0.91	(0.82, 0.96)	0.87	(0.82, 1.01)	0.845
Taurine	2.53	(1.73, 3.81)	2.65	(1.75, 3.63)	0.876
Serine	1.33	(1.21, 1.52)	1.35	(1.22, 1.50)	0.607
Glycine	1.50	(1.32, 1.71)	1.52	(1.29, 1.65)	0.760
Riboflavin	5.39	(3.40, 9.56)	6.07	(3.36, 9.20)	0.961
Pyridoxamine	1.24	(1.00, 1.86)	1.40	(1.08, 1.78)	0.408
Pyridoxine	1.71	(1.09, 3.05)	2.11	(1.21, 4.44)	0.355

^a^ The difference in cord blood/maternal blood at birth ratio between the FA non-user group and the FA user group was tested using the Mann–Whitney U test.

## Data Availability

The raw data supporting the conclusions of this article will be made available by the authors upon request.

## Supplementary Materials

The following supporting information can be downloaded at: https://www.mdpi.com/article/10.3390/metabo16040215/s1, Figure S1: Scatter plot of serum 5-MTHF concentration in maternal blood at birth versus serum 5-MTHF concentration in cord blood; Figure S2. Scatter plot of serum 5-MTHF concentration in maternal blood at birth versus serum total homocysteine concentration in maternal blood at birth; Table S1. Energy intake defining underreporting (kcal/day); Table S2. Previous studies investigating 5-MTHF and FA; Table S3. Correlation between FA intake and OCM-related metabolites.

## Abbreviations

The following abbreviations are used in this manuscript:

5-MTHF	5-methyl tetrahydrofolate
BDHQ	Brief-type self-administered diet history questionnaire
BHMT	Betaine–homocysteine methyltransferase
BMI	Body mass index
C-MACH	Chiba study of Mother and Child Health
DMG	Dimethylglycine
DOHaD	Developmental Origins of Health and Disease
FA	Folic acid
Hcy	Homocysteine
LC-MS/MS	Liquid chromatography-tandem mass spectrometry
MS	Methionine synthase
NCDs	Non-communicable diseases
OCM	One-carbon metabolism
SAH	*S*-adenosylhomocysteine
SAM	*S*-adenosylmethionine
SHMT	Serine hydroxymethyltransferase
tCys	Total cysteine
tHcy	Total homocysteine
UMFA	Unmetabolized folic acid

## References

[1] Steegers-Theunissen R.P., Twigt J., Pestinger V., Sinclair K.D. (2013). The periconceptional period, reproduction and long-term health of offspring: The importance of one-carbon metabolism. Hum. Reprod. Update.

[2] Godfrey K.M., Lillycrop K.A., Burdge G.C., Gluckman P.D., Hanson M.A. (2007). Epigenetic mechanisms and the mismatch concept of the developmental origins of health and disease. Pediatr. Res..

[3] Waterland R.A., Michels K.B. (2007). Epigenetic epidemiology of the developmental origins hypothesis. Annu. Rev. Nutr..

[4] Reik W., Walter J. (2001). Genomic imprinting: Parental influence on the genome. Nat. Rev. Genet..

[5] Bortz J., Obeid R. (2025). The Shuttling of Methyl Groups Between Folate and Choline Pathways. Nutrients.

[6] Zeisel S.H. (2009). Importance of methyl donors during reproduction. Am. J. Clin. Nutr..

[7] Tibbetts A.S., Appling D.R. (2010). Compartmentalization of Mammalian folate-mediated one-carbon metabolism. Annu. Rev. Nutr..

[8] Hoffbrand A.V., Weir D.G. (2001). The history of folic acid. Br. J. Haematol..

[9] Stover P.J., Field M.S. (2011). Trafficking of intracellular folates. Adv. Nutr..

[10] Fox J.T., Stover P.J. (2008). Folate-mediated one-carbon metabolism. Vitam. Horm..

[11] Clare C.E., Brassington A.H., Kwong W.Y., Sinclair K.D. (2019). One-Carbon Metabolism: Linking Nutritional Biochemistry to Epigenetic Programming of Long-Term Development. Annu. Rev. Anim. Biosci..

[12] Kubo Y., Fukuoka H., Kawabata T., Shoji K., Mori C., Sakurai K., Nishikawa M., Ohkubo T., Oshida K., Yanagisawa N. (2020). Distribution of 5-Methyltetrahydrofolate and Folic Acid Levels in Maternal and Cord Blood Serum: Longitudinal Evaluation of Japanese Pregnant Women. Nutrients.

[13] Kubo Y., Fukuoka H., Shoji K., Mori C., Sakurai K., Nishikawa M., Oshida K., Yamashiro Y., Kawabata T. (2024). Longitudinal Analysis of One-Carbon Metabolism-Related Metabolites in Maternal and Cord Blood of Japanese Pregnant Women. Nutrients.

[14] Kubo Y., Shoji K., Tajima A., Horiguchi S., Fukuoka H., Nishikawa M., Kagawa Y., Kawabata T. (2023). Serum 5-Methyltetrahydrofolate Status Is Associated with One-Carbon Metabolism-Related Metabolite Concentrations and Enzyme Activity Indicators in Young Women. Int. J. Mol. Sci..

[15] Scaglione F., Panzavolta G. (2014). Folate, folic acid and 5-methyltetrahydrofolate are not the same thing. Xenobiotica.

[16] Saini R.K., Nile S.H., Keum Y.S. (2016). Folates: Chemistry, analysis, occurrence, biofortification and bioavailability. Food Res. Int..

[17] Delchier N., Herbig A.-L., Rychlik M., Renard C.M.G.C. (2016). Folates in Fruits and Vegetables: Contents, Processing, and Stability. Compr. Rev. Food Sci. Food Saf..

[18] Konings E.J., Roomans H.H., Dorant E., Goldbohm R.A., Saris W.H., van den Brandt P.A. (2001). Folate intake of the Dutch population according to newly established liquid chromatography data for foods. Am. J. Clin. Nutr..

[19] Visentin M., Diop-Bove N., Zhao R., Goldman I.D. (2014). The intestinal absorption of folates. Annu. Rev. Physiol..

[20] Melse-Boonstra A., Verhoef P., Konings E.J., Van Dusseldorp M., Matser A., Hollman P.C., Meyboom S., Kok F.J., West C.E. (2002). Influence of processing on total, monoglutamate and polyglutamate folate contents of leeks, cauliflower, and green beans. J. Agric. Food Chem..

[21] Indrawati, Arroqui C., Messagie I., Nguyen M.T., Van Loey A., Hendrickx M. (2004). Comparative study on pressure and temperature stability of 5-methyltetrahydrofolic acid in model systems and in food products. J. Agric. Food Chem..

[22] Caudill M.A. (2010). Folate bioavailability: Implications for establishing dietary recommendations and optimizing status. Am. J. Clin. Nutr..

[23] Mnkeni A.P., Beveridge T. (1982). Thermal Destruction of Pteroylglutamic Acid in Buffer and Model Food Systems. J. Food Sci..

[24] Paine-Wilson B., Chen T.S. (1979). Thermal destruction of folacin: Effect of pH and buffer ions. J. Food Sci..

[25] Steindal A.H., Juzeniene A., Johnsson A., Moan J. (2006). Photodegradation of 5-methyltetrahydrofolate: Biophysical aspects. Photochem. Photobiol..

[26] Melse-Boonstra A., Verhoef P., West C. (2004). Quantifying folate bioavailability: A critical appraisal of methods. Curr. Opin. Clin. Nutr. Metab. Care.

[27] Ohrvik V.E., Witthoft C.M. (2011). Human folate bioavailability. Nutrients.

[28] Berti C., Fekete K., Dullemeijer C., Trovato M., Souverein O.W., Cavelaars A., Dhonukshe-Rutten R., Massari M., Decsi T., Van’t Veer P. (2012). Folate intake and markers of folate status in women of reproductive age, pregnant and lactating women: A meta-analysis. J. Nutr. Metab..

[29] West A.A., Yan J., Perry C.A., Jiang X., Malysheva O.V., Caudill M.A. (2012). Folate-status response to a controlled folate intake in nonpregnant, pregnant, and lactating women. Am. J. Clin. Nutr..

[30] Plumptre L., Masih S.P., Ly A., Aufreiter S., Sohn K.J., Croxford R., Lausman A.Y., Berger H., O’Connor D.L., Kim Y.I. (2015). High concentrations of folate and unmetabolized folic acid in a cohort of pregnant Canadian women and umbilical cord blood. Am. J. Clin. Nutr..

[31] Obeid R., Kasoha M., Kirsch S.H., Munz W., Herrmann W. (2010). Concentrations of unmetabolized folic acid and primary folate forms in pregnant women at delivery and in umbilical cord blood. Am. J. Clin. Nutr..

[32] McNulty B., McNulty H., Marshall B., Ward M., Molloy A.M., Scott J.M., Dornan J., Pentieva K. (2013). Impact of continuing folic acid after the first trimester of pregnancy: Findings of a randomized trial of Folic Acid Supplementation in the Second and Third Trimesters. Am. J. Clin. Nutr..

[33] Fernandez-Roig S., Cavalle-Busquets P., Fernandez-Ballart J.D., Ballesteros M., Berrocal-Zaragoza M.I., Salat-Batlle J., Ueland P.M., Murphy M.M. (2013). Low folate status enhances pregnancy changes in plasma betaine and dimethylglycine concentrations and the association between betaine and homocysteine. Am. J. Clin. Nutr..

[34] Stam F., Smulders Y.M., van Guldener C., Jakobs C., Stehouwer C.D., de Meer K. (2005). Folic acid treatment increases homocysteine remethylation and methionine transmethylation in healthy subjects. Clin. Sci..

[35] Steegers-Theunissen R.P., Obermann-Borst S.A., Kremer D., Lindemans J., Siebel C., Steegers E.A., Slagboom P.E., Heijmans B.T. (2009). Periconceptional maternal folic acid use of 400 microg per day is related to increased methylation of the IGF2 gene in the very young child. PLoS ONE.

[36] Sakurai K., Miyaso H., Eguchi A., Matsuno Y., Yamamoto M., Todaka E., Fukuoka H., Hata A., Mori C., Chiba Study of Mother and Children’s Health Group (2016). Chiba study of Mother and Children’s Health (C-MACH): Cohort study with omics analyses. BMJ Open.

[37] Bailey R.L., Dodd K.W., Gahche J.J., Dwyer J.T., McDowell M.A., Yetley E.A., Sempos C.A., Burt V.L., Radimer K.L., Picciano M.F. (2010). Total folate and folic acid intake from foods and dietary supplements in the United States: 2003–2006. Am. J. Clin. Nutr..

[38] Kobayashi S., Honda S., Murakami K., Sasaki S., Okubo H., Hirota N., Notsu A., Fukui M., Date C. (2012). Both comprehensive and brief self-administered diet history questionnaires satisfactorily rank nutrient intakes in Japanese adults. J. Epidemiol..

[39] Ministry of Health, Labour and Welfare (2015). Dietary Reference Intakes for Japanese, 2015.

[40] Shiraishi M., Haruna M., Matsuzaki M., Murayama R., Sasaki S. (2015). The biomarker-based validity of a brief-type diet history questionnaire for estimating eicosapentaenoic acid and docosahexaenoic acid intakes in pregnant Japanese women. Asia Pac. J. Clin. Nutr..

[41] Ishikawa T., Obara T., Nishigori H., Nishigori T., Metoki H., Ishikuro M., Tatsuta N., Mizuno S., Sakurai K., Nishijima I. (2020). Update on the prevalence and determinants of folic acid use in Japan evaluated with 91,538 pregnant women: The Japan Environment and Children’s Study. J. Matern. Fetal Neonatal Med..

[42] Saragih I.D., Dimog E.F., Saragih I.S., Lin C.J. (2022). Adherence to Iron and Folic Acid Supplementation (IFAS) intake among pregnant women: A systematic review meta-analysis. Midwifery.

[43] Zheng W., Zhang Y., Zhang P., Chen T., Yan X., Li L., Shao L., Song Z., Han W., Wang J. (2024). Gestational diabetes mellitus is associated with distinct folate-related metabolites in early and mid-pregnancy: A prospective cohort study. Diabetes Metab. Res. Rev..

[44] Maulik D., van Haandel L., Allsworth J., Chaisanguanthum K.S., Yeast J.D., Leeder J.S. (2021). The effect of race and supplementation on maternal and umbilical cord plasma folates. J. Matern. Fetal Neonatal Med..

[45] Stark K.D., Pawlosky R.J., Sokol R.J., Hannigan J.H., Salem N. (2007). Maternal smoking is associated with decreased 5-methyltetrahydrofolate in cord plasma. Am. J. Clin. Nutr..

[46] Stark K.D., Pawlosky R.J., Beblo S., Murthy M., Flanagan V.P., Janisse J., Buda-Abela M., Rockett H., Whitty J.E., Sokol R.J. (2005). Status of plasma folate after folic acid fortification of the food supply in pregnant African American women and the influences of diet, smoking, and alcohol consumption. Am. J. Clin. Nutr..

[47] Raghavan R., Selhub J., Paul L., Ji Y., Wang G., Hong X., Zuckerman B., Fallin M.D., Wang X. (2020). A prospective birth cohort study on cord blood folate subtypes and risk of autism spectrum disorder. Am. J. Clin. Nutr..

[48] Bodnar L.M., Himes K.P., Venkataramanan R., Chen J.Y., Evans R.W., Meyer J.L., Simhan H.N. (2010). Maternal serum folate species in early pregnancy and risk of preterm birth. Am. J. Clin. Nutr..

[49] Sweeney M.R., McPartlin J., Weir D.G., Daly S., Pentieva K., Daly L., Scott J.M. (2005). Evidence of unmetabolised folic acid in cord blood of newborn and serum of 4-day-old infants. Br. J. Nutr..

[50] Cochrane K.M., Elango R., Devlin A.M., Mayer C., Hutcheon J.A., Karakochuk C.D. (2024). Supplementation with (6S)-5-methyltetrahydrofolic acid appears as effective as folic acid in maintaining maternal folate status while reducing unmetabolised folic acid in maternal plasma: A randomised trial of pregnant women in Canada. Br. J. Nutr..

[51] Sulistyoningrum D.C., Sullivan T.R., Skubisz M., Palmer D.J., Wood S., Ueland P.M., McCann A., Makrides M., Green T.J., Best K.P. (2024). Maternal serum unmetabolized folic acid concentration following multivitamin and mineral supplementation with or without folic acid after 12 weeks gestation: A randomized controlled trial. Matern. Child. Nutr..

[52] Murphy M.S.Q., Muldoon K.A., Sheyholislami H., Behan N., Lamers Y., Rybak N., White R.R., Harvey A.L.J., Gaudet L.M., Smith G.N. (2021). Impact of high-dose folic acid supplementation in pregnancy on biomarkers of folate status and 1-carbon metabolism: An ancillary study of the Folic Acid Clinical Trial (FACT). Am. J. Clin. Nutr..

[53] Plumptre L., Tammen S.A., Sohn K.J., Masih S.P., Visentin C.E., Aufreiter S., Malysheva O., Schroder T.H., Ly A., Berger H. (2020). Maternal and Cord Blood Folate Concentrations Are Inversely Associated with Fetal DNA Hydroxymethylation, but Not DNA Methylation, in a Cohort of Pregnant Canadian Women. J. Nutr..

[54] Wright A.J., Finglas P.M., Dainty J.R., Wolfe C.A., Hart D.J., Wright D.M., Gregory J.F. (2005). Differential kinetic behavior and distribution for pteroylglutamic acid and reduced folates: A revised hypothesis of the primary site of PteGlu metabolism in humans. J. Nutr..

[55] Patanwala I., King M.J., Barrett D.A., Rose J., Jackson R., Hudson M., Philo M., Dainty J.R., Wright A.J., Finglas P.M. (2014). Folic acid handling by the human gut: Implications for food fortification and supplementation. Am. J. Clin. Nutr..

[56] Nijhout H.F., Reed M.C., Lam S.L., Shane B., Gregory J.F., Ulrich C.M. (2006). In silico experimentation with a model of hepatic mitochondrial folate metabolism. Theor. Biol. Med. Model..

[57] Holm P.I., Ueland P.M., Vollset S.E., Midttun O., Blom H.J., Keijzer M.B., den Heijer M. (2005). Betaine and folate status as cooperative determinants of plasma homocysteine in humans. Arterioscler. Thromb. Vasc. Biol..

[58] Dasarathy J., Gruca L.L., Bennett C., Parimi P.S., Duenas C., Marczewski S., Fierro J.L., Kalhan S.C. (2010). Methionine metabolism in human pregnancy. Am. J. Clin. Nutr..

[59] Lee H.S. (2015). Impact of Maternal Diet on the Epigenome during In Utero Life and the Developmental Programming of Diseases in Childhood and Adulthood. Nutrients.

[60] Pauwels S., Ghosh M., Duca R.C., Bekaert B., Freson K., Huybrechts I., Langie S.A.S., Koppen G., Devlieger R., Godderis L. (2017). Maternal intake of methyl-group donors affects DNA methylation of metabolic genes in infants. Clin. Epigenet..

[61] Fryer A.A., Nafee T.M., Ismail K.M., Carroll W.D., Emes R.D., Farrell W.E. (2009). LINE-1 DNA methylation is inversely correlated with cord plasma homocysteine in man: A preliminary study. Epigenetics.

[62] Levonen A.L., Lapatto R., Saksela M., Raivio K.O. (2000). Human cystathionine gamma-lyase: Developmental and in vitro expression of two isoforms. Biochem. J..

[63] Gaull G., Sturman J.A., Raiha N.C. (1972). Development of mammalian sulfur metabolism: Absence of cystathionase in human fetal tissues. Pediatr. Res..

[64] Heinonen K., Raiha N.C. (1974). Induction of cystathionase in human foetal liver. Biochem. J..

[65] Shew S.B., Keshen T.H., Jahoor F., Jaksic T. (2005). Assessment of cysteine synthesis in very low-birth weight neonates using a [13C6]glucose tracer. J. Pediatr. Surg..

[66] Sturman J.A., Gaull G., Raiha N.C. (1970). Absence of cystathionase in human fetal liver: Is cystine essential?. Science.

[67] Vina J., Vento M., Garcia-Sala F., Puertes I.R., Gasco E., Sastre J., Asensi M., Pallardo F.V. (1995). L-cysteine and glutathione metabolism are impaired in premature infants due to cystathionase deficiency. Am. J. Clin. Nutr..

[68] Ripps H., Shen W. (2012). Review: Taurine: A “very essential” amino acid. Mol. Vis..

[69] Kilb W., Fukuda A. (2017). Taurine as an Essential Neuromodulator during Perinatal Cortical Development. Front. Cell Neurosci..

[70] Dawson P.A. (2011). Sulfate in fetal development. Semin. Cell Dev. Biol..

[71] Dawson P.A., Richard K., Perkins A., Zhang Z., Simmons D.G. (2017). Review: Nutrient sulfate supply from mother to fetus: Placental adaptive responses during human and animal gestation. Placenta.

[72] Livingstone M.B., Black A.E. (2003). Markers of the validity of reported energy intake. J. Nutr..

[73] McGowan C.A., McAuliffe F.M. (2012). Maternal nutrient intakes and levels of energy underreporting during early pregnancy. Eur. J. Clin. Nutr..

[74] Willett W.C., Howe G.R., Kushi L.H. (1997). Adjustment for total energy intake in epidemiologic studies. Am. J. Clin. Nutr..

[75] Fredriksen A., Meyer K., Ueland P.M., Vollset S.E., Grotmol T., Schneede J. (2007). Large-scale population-based metabolic phenotyping of thirteen genetic polymorphisms related to one-carbon metabolism. Hum. Mutat..

[76] Pfeiffer C.M., Sternberg M.R., Fazili Z., Yetley E.A., Lacher D.A., Bailey R.L., Johnson C.L. (2015). Unmetabolized folic acid is detected in nearly all serum samples from US children, adolescents, and adults. J. Nutr..

[77] Hao L., Yang Q.H., Li Z., Bailey L.B., Zhu J.H., Hu D.J., Zhang B.L., Erickson J.D., Zhang L., Gindler J. (2008). Folate status and homocysteine response to folic acid doses and withdrawal among young Chinese women in a large-scale randomized double-blind trial. Am. J. Clin. Nutr..

[78] Crider K.S., Devine O., Qi Y.P., Yeung L.F., Sekkarie A., Zaganjor I., Wong E., Rose C.E., Berry R.J. (2019). Systematic Review and Bayesian Meta-analysis of the Dose-response Relationship between Folic Acid Intake and Changes in Blood Folate Concentrations. Nutrients.

