# Supplementary material for: Association Between Folic Acid Use and Serum One-Carbon Metabolism-Related Metabolites in Maternal and Cord Blood of Japanese Pregnant Women
Source: Metabolites. 2026 Mar 25;16(4):215. doi: 10.3390/metabo16040215 (PMC13118084; doi:10.3390/metabo16040215)
Supplement: Supplementary file 1 [file metabolites-16-00215-s001.zip › metabolites-4157200-supplementary.pdf]

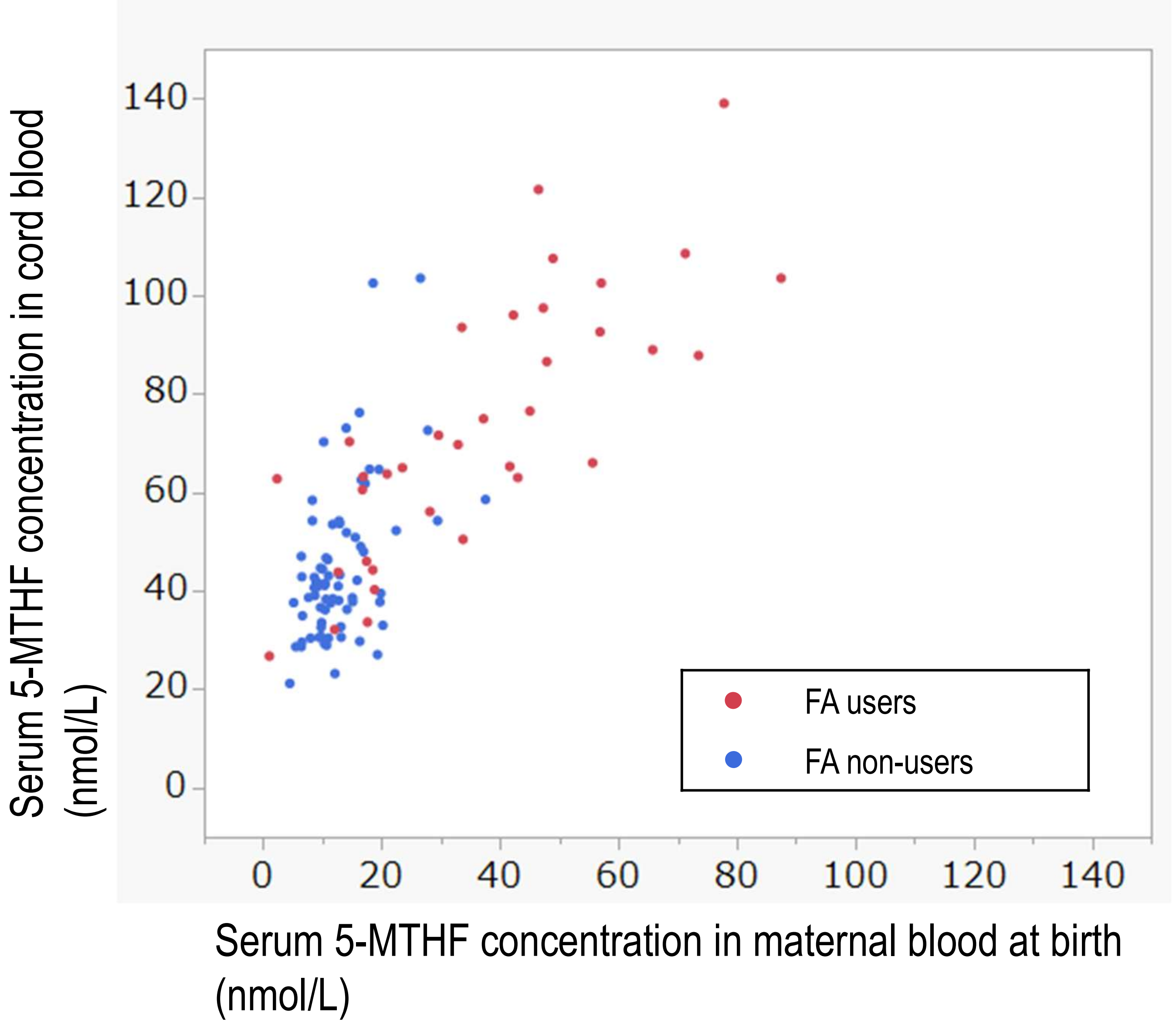


**Figure S1.** Scatter plot of serum 5-MTHF concentration in maternal blood at birth versus serum 5-MTHF concentration in cord blood. Red dots indicate FA users, while blue dots indicate FA non-users.

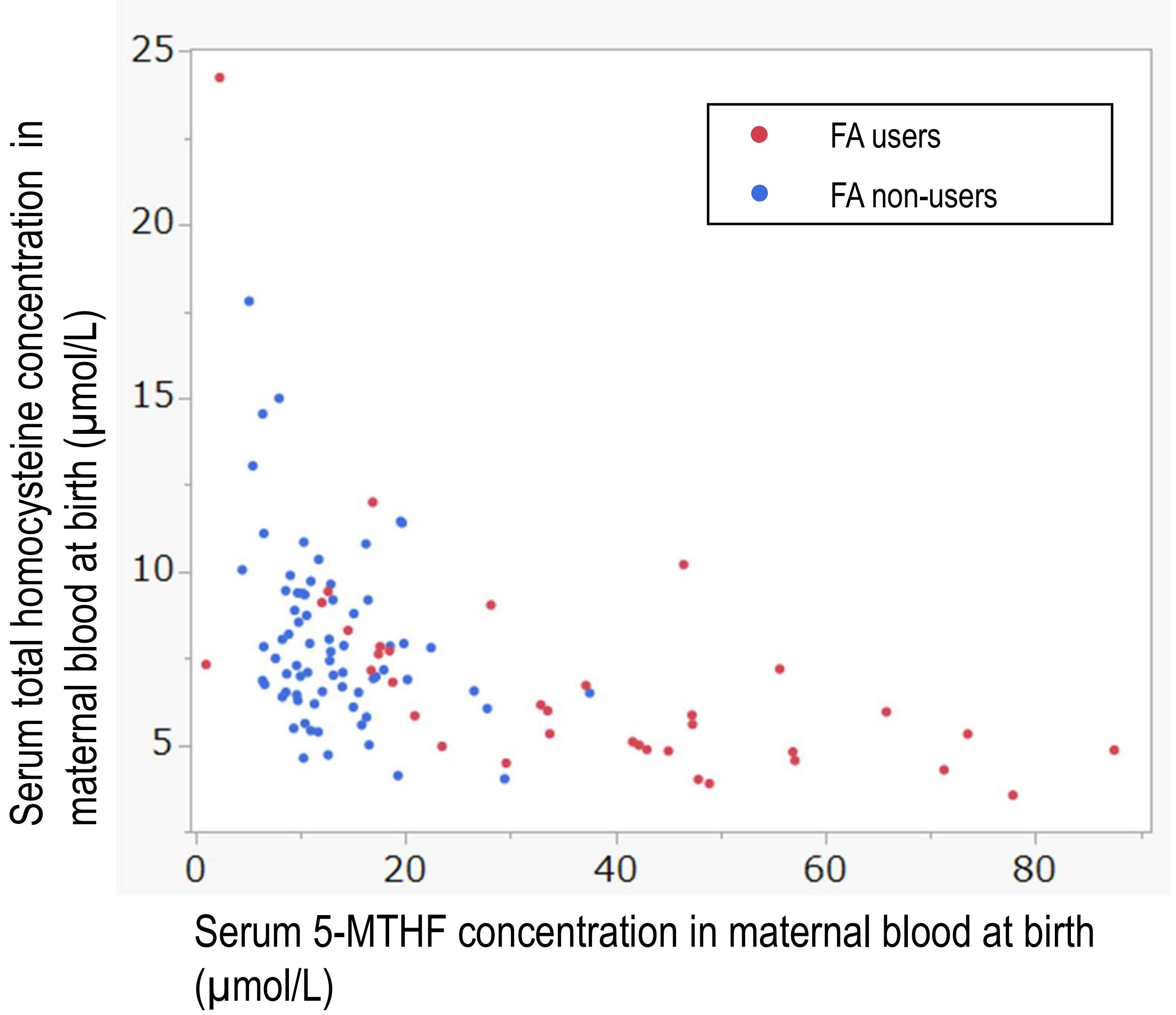


**Figure S2.** Scatter plot of serum 5-MTHF concentration in maternal blood at birth versus serum total homocysteine concentration in maternal blood at birth. Red dots indicate FA users, while blue dots indicate FA non-users.

**Table S1.** Energy intake defining underreporting (kcal/day).

| Energy (kcal/day) | Early pregnancy | | Late pregnancy | |
|---|---|---|---|---|
| | Underreported | Overreported | Underreported | Overreported |
| Women aged 18–29 | < 850 | > 3825 | < 1050 | > 3995 |
| Women aged 30–49 | < 900 | > 4505 | < 1100 | > 4675 |

**Table S2.** Previous studies investigating 5-MTHF and FA.

| Paper Information | | | Characteristics | | | | | | 5-MTHF (nmol/L) | | | UMFA (nmol/L) | | |
|---|---|---|---|---|---|---|---|---|---|---|---|---|---|---|
| First author | Publication Year | References cited in the main text | Samples | | Countries | Mandatory FA Fortification | Gestational week | Ranges | Representative values | Ranges | Values are expressed as | Representative values | Ranges | Values are expressed as |
| Cochrane KM | 2024 | 50 | Maternal blood | Plasma | Canada | Yes<br>Baseline for the intervention trial | 18 | 13-20 | - | - | - | 1.1 | 0.9-1.5 | Median; IQR |
| Kubo Y | 2024 | 13 | Maternal blood | Serum | Japan | No | 11.4 | ±0.8 | 32.2 | 20.3-52.8 | Median; IQR | 0.62 | 0.10-1.22 | Median; IQR |
| Kubo Y | 2024 | 13 | Maternal blood | Serum | Japan | No | 28.4 | ±1.0 | 17.0 | 11.6-31.7 | Median; IQR | 0.62 | 0.13-1.21 | Median; IQR |
| Kubo Y | 2024 | 13 | Maternal blood | Serum | Japan | No | 39.5 | ±1.1 | 14.1 | 9.8-23.2 | Median; IQR | 0.43 | 0-1.01 | Median; IQR |
| Sulistyoningrum DC | 2024 | 51 | Maternal blood | Serum | Australia | Yes<br>After intervention (0.8 mg/day for 12–16 weeks up to 36 weeks) | 36 | なし | - | - | - | 1.4 | ±2.7 | Mean; SD. |
| Zheng W | 2024 | 43 | Maternal blood | Plasma | China | No | 10 | 8.1-16.0 | 22.5 | 16.3-29.9 | Median; IQR | - | - | - |
| Zheng W | 2024 | 43 | Maternal blood | Plasma | China | No | 22.9 | 20.4–25.1 | 23.4 | 16.2-34.3 | Median; IQR | - | - | - |
| Maulik D | 2021 | 44 | Maternal blood | Plasma | United States | Yes | 39.0 | ±1.8 | 39.1 | ±18.8 | Mean; SD. | 3.3 | ±9.5 | Mean; SD. |
| Murphy MSQ | 2021 | 52 | Maternal blood | Serum | Canada | Yes<br>After the intervention (1 mg/day × 10.9 weeks) | 25.3 | 24.3-26.1 | 108.6 | 96.4-123.2 | Median; IQR | 1.9 | 0.9-4.1 | Median; IQR |
| Plumptre L | 2020 | 53 | Maternal blood | Plasma | Canada | Yes | 14 | 12-16 | - | - | - | 1.04 | 1.03-1.04 | Mean; 95% CI |
| Plumptre L | 2015 | 30 | Maternal blood | Plasma | Canada | Yes | 13.1 | 13.0-13.3 | - | - | - | 2.41 | 1.99-2.88 | Mean; 95% CI |
| West AA | 2012 | 29 | Maternal blood | Serum | United States | Yes | 27 | ≤ 27 | 65.3 | 53.1-75.5 | Median; 95% CI | 0.93 | 0.23-1.47 | Median; 95% CI |
| Bodnar LM | 2010 | 48 | Maternal blood | Serum | United States | Yes | 9.4 | 7.5-12.1 | 34.4 | 25.2-47.7 | Median; IQR | - | - | - |
| Obeid R | 2010 | 31 | Maternal blood | Serum | Germany | No | 39 | 36.0-40.2 | 15.0 | 4.0-41.9 | Median; 10th-90th percentile | 0.15 | 0.00-0.80 | Median; 10th-90th percentile |
| Stark KD | 2007 | 45 | Maternal blood | Plasma | United States | Yes | 24 | - | 28.3 | ±16.3 | Mean; SD. | - | - | - |
| Stark KD | 2005 | 46 | Maternal blood | Plasma | United States | Yes | 24 | - | 39.2 | ±15.5 | Mean; SD. | - | - | - |
| Kubo Y | 2024 | 13 | Cord blood | Serum | Japan | No | - | - | 44.7 | 36.5-64.2 | Median; IQR | 0.53 | 0-1.00 | Median; IQR |
| Maulik D | 2021 | 44 | Cord blood | Plasma | United States | Yes | - | - | 59.5 | ±21.9 | Mean; SD. | 0.40 | ±0.4 | Mean; SD. |
| Plumptre L | 2020 | 53 | Cord blood | Plasma | Canada | Yes | - | - | - | - | - | 1.03 | 1.03-1.03 | Mean; 95% CI |
| Raghavan R | 2020 | 47 | Cord blood | Plasma | United States | Yes | - | - | 52.4 | ±33.6 | Mean; SD. | 1.24 | ±2.7 | Mean; SD. |
| Plumptre L | 2015 | 30 | Cord blood | Plasma | Canada | Yes | - | - | - | - | - | 0.68 | 0.60-0.77 | Mean; 95% CI |
| Obeid R | 2010 | 31 | Cord blood | Serum | Germany | No | - | - | 37.8 | 7.7-76.5 | Median; 10th-90th percentile | 0.21 | 0.00-0.51 | Median; 10th-90th percentile |
| Stark KD | 2007 | 45 | Cord blood | Plasma | United States | Yes | - | - | 36.6 | ±16.3 | Mean; SD. | - | - | - |
| Sweeney MR | 2005 | 49 | Cord blood | Serum | Ireland | No | - | - | - | - | - | 0.419 | ±0.122 | Mean; SD. |

**Table S3.** Correlation between FA intake and OCM-related metabolites.

| OCM-Related Metabolite Concentrations | Maternal blood | | | | | | Cord Blood | |
|---|---|---|---|---|---|---|---|---|
| | Early Pregnancy $n$ = 130 | | Late Pregnancy $n$ = 116 | | At delivery $n$ = 108 | | $n$ = 113 | |
| | ρ | $p$-value | ρ | $p$-value | ρ | $p$-value | ρ | $p$-value |
| 5-MTHF | 0.767 | <.0001 | 0.607 | <.0001 | 0.615 | <.0001 | 0.545 | <.0001 |
| UMFA | 0.196 | 0.026 | 0.155 | 0.097 | 0.103 | 0.291 | -0.002 | 0.986 |
| Choline | 0.125 | 0.156 | 0.040 | 0.670 | -0.038 | 0.695 | -0.018 | 0.854 |
| Betaine | 0.031 | 0.726 | 0.206 | 0.026 | 0.149 | 0.125 | 0.091 | 0.341 |
| DMG | -0.032 | 0.720 | -0.013 | 0.886 | -0.149 | 0.124 | -0.185 | 0.050 |
| Betaine/DMG | 0.036 | 0.689 | 0.086 | 0.358 | 0.241 | 0.012 | 0.172 | 0.068 |
| Methionine | 0.126 | 0.152 | 0.104 | 0.269 | 0.049 | 0.615 | 0.224 | 0.017 |
| SAM | 0.206 | 0.019 | 0.235 | 0.011 | 0.126 | 0.193 | 0.187 | 0.047 |
| SAH | 0.053 | 0.547 | 0.049 | 0.605 | 0.127 | 0.192 | 0.107 | 0.258 |
| SAM/SAH | 0.076 | 0.393 | 0.085 | 0.365 | -0.056 | 0.567 | -0.030 | 0.757 |
| Homocysteine | -0.205 | 0.019 | -0.249 | 0.007 | -0.307 | 0.001 | -0.326 | <.001 |
| Cystathionine | -0.026 | 0.766 | -0.059 | 0.529 | -0.177 | 0.066 | -0.013 | 0.889 |
| Cysteine | 0.158 | 0.074 | 0.279 | 0.002 | 0.149 | 0.123 | 0.201 | 0.033 |
| Homocystein/Cysteine | -0.339 | <.0001 | -0.395 | <.0001 | -0.433 | <.0001 | -0.397 | <.0001 |
| Taurine | 0.146 | 0.098 | -0.078 | 0.409 | 0.067 | 0.494 | -0.040 | 0.678 |
| Serine | -0.134 | 0.130 | -0.070 | 0.459 | -0.082 | 0.397 | 0.052 | 0.582 |
| Glycine | 0.134 | 0.127 | 0.131 | 0.162 | 0.069 | 0.481 | 0.110 | 0.248 |
| Serine/Glycine | -0.302 | <.001 | -0.215 | 0.021 | -0.112 | 0.250 | -0.079 | 0.408 |
| Riboflavin | 0.191 | 0.030 | 0.249 | 0.007 | 0.277 | 0.004 | 0.199 | 0.035 |
| Pyridoxamine | 0.043 | 0.628 | 0.141 | 0.132 | 0.159 | 0.100 | 0.258 | 0.006 |
| Pyridoxine | 0.174 | 0.048 | 0.107 | 0.255 | 0.037 | 0.707 | 0.129 | 0.175 |

ρ：Spearman correlation coefficient

**Table S4.** Comparison of serum OCM-related metabolite concentrations in maternal blood and umbilical cord blood during early pregnancy, late pregnancy, and delivery, stratified by FA intake category.

| | Analytes and Metabolic Indicators | Samples/period | FA non-users | | FA users * | | | | | |
|---|---|---|---|---|---|---|---|---|---|---|
| | | | 0 μg/day | | >0 to <400 μg/day | | 400 μg/day | | >400 μg/day | |
| | | | *n* | Median (25th, 75th) | *n* | Median (25th, 75th) | *n* | Median (25th, 75th) | *n* | Median (25th, 75th) |
| FA intake (μg/day) | | Early pregnancy | 59 | 0 (0, 0) $^{a}$ | 30 | 207 (143, 289) $^{b}$ | 21 | 400 (400, 400) $^{c}$ | 20 | 483 (480, 769) $^{d}$ |
| | | Late pregnancy | 81 | 0 (0, 0) $^{a}$ | 14 | 143 (114, 200) $^{b}$ | 15 | 400 (400, 400) $^{c}$ | 10 | 480 (478, 505) $^{d}$ |
| Folate Cycle | 5-MTHF, nmol/L | Maternal blood in early pregnancy | 59 | 19.8 (16.2, 27.5) $^{a}$ | 30 | 42.3 (26.3, 56.2) $^{b}$ | 21 | 56.3 (40.6, 66.5) $^{b}$ | 20 | 50.2 (40.3, 57.6) $^{b}$ |
| | | Maternal blood in late pregnancy | 78 | 13.7 (10.8, 19.2) $^{a}$ | 14 | 34.1 (22.1, 48.5) $^{b}$ | 15 | 57.1 (36.5, 69.1) $^{b}$ | 9 | 54.4 (23.9, 67.9) $^{b}$ |
| | | Maternal blood at delivery | 72 | 11.2 (9.4, 16.2) $^{a}$ | 12 | 28.5 (15.6, 44.5) $^{b}$ | 15 | 42.3 (28.2, 57.1) $^{b}$ | 9 | 29.6 (16.8, 52.1) $^{b}$ |
| | | Cord blood | 76 | 41.0 (33.1, 51.6) $^{a}$ | 14 | 63.4 (47.3, 80.8) $^{b}$ | 15 | 75.0 (62.8, 96.0) $^{b}$ | 8 | 67.4 (48.3, 96.2) $^{b}$ |
| | UMFA, nmol/L | Maternal blood in early pregnancy | 59 | 0.545 (0.000, 0.910) | 30 | 0.582 (0.073, 1.671) | 21 | 1.035 (0.487, 1.890) | 20 | 0.832 (0.031, 1.691) |
| | | Maternal blood in late pregnancy | 78 | 0.599 (0.000, 1.195) | 14 | 0.663 (0.283, 1.020) | 15 | 0.818 (0.435, 2.220) | 9 | 0.510 (0.235, 1.542) |
| | | Maternal blood at delivery | 72 | 0.434 (0.000, 0.799) | 12 | 0.286 (0.000, 1.315) | 15 | 0.378 (0.000, 1.610) | 9 | 0.600 (0.433, 1.995) |
| | | Cord blood | 76 | 0.530 (0.139, 1.098) | 14 | 0.685 (0.000, 1.018) | 15 | 0.855 (0.134, 1.429) | 8 | 0.340 (0.000, 0.676) |
| Choline Metabolic Pathway | Choline, μmol/L | Maternal blood in early pregnancy | 59 | 6.94 (6.05, 8.84) | 30 | 7.35 (6.06, 8.96) | 21 | 8.73 (6.90, 9.43) | 20 | 7.28 (6.75, 8.64) |
| | | Maternal blood in late pregnancy | 78 | 7.78 (6.88, 9.73) | 14 | 9.26 (6.74, 10.36) | 15 | 8.91 (6.82, 9.77) | 9 | 6.84 (6.69, 11.90) |
| | | Maternal blood at delivery | 72 | 11.58 (9.96, 12.76) | 12 | 12.10 (9.02, 14.96) | 15 | 11.00 (9.45, 12.35) | 9 | 9.93 (8.62, 16.15) |
| | | Cord blood | 76 | 28.70 (25.15, 31.79) | 14 | 29.93 (25.58, 34.33) | 15 | 25.55 (22.55, 27.75) | 8 | 30.50 (27.99, 42.70) |
| | Betaine, μmol/L | Maternal blood in early pregnancy | 59 | 21.4 (17.0, 25.5) | 30 | 21.4 (16.7, 24.9) | 21 | 21.3 (17.0, 30.5) | 20 | 21.1 (18.3, 25.8) |
| | | Maternal blood in late pregnancy | 78 | 13.1 (11.7, 15.2) | 14 | 14.9 (13.5, 18.4) | 15 | 17.6 (11.5, 22.4) | 9 | 14.6 (11.3, 18.2) |
| | | Maternal blood at delivery | 72 | 13.5 (11.8, 15.7) | 12 | 14.2 (11.3, 16.2) | 15 | 14.6 (11.3, 17.6) | 9 | 15.9 (12.2, 19.6) |
| | | Cord blood | 76 | 26.4 (23.4, 31.5) | 14 | 28.9 (24.9, 32.0) | 15 | 26.8 (25.0, 27.9) | 8 | 30.7 (24.3, 37.9) |
| | DMG, μmol/L | Maternal blood in early pregnancy | 59 | 1.83 (1.24, 2.54) | 30 | 1.50 (0.90, 2.49) | 21 | 2.05 (1.45, 2.50) | 20 | 1.61 (1.28, 2.01) |
| | | Maternal blood in late pregnancy | 78 | 1.57 (1.09, 2.45) | 14 | 1.62 (0.91, 2.36) | 15 | 1.61 (1.21, 2.00) | 9 | 1.90 (1.10, 2.75) |
| | | Maternal blood at delivery | 72 | 2.38 (1.72, 3.75) | 12 | 2.05 (1.42, 3.38) | 15 | 1.70 (1.32, 2.44) | 9 | 1.99 (1.76, 4.04) |
| | | Cord blood | 76 | 3.33 (2.68, 4.21) $^{a}$ | 14 | 3.04 (2.18, 3.75) | 15 | 2.60 (1.67, 3.46) $^{b}$ | 8 | 3.06 (2.86, 5.25) |
| | Betaine/DMG | Maternal blood in early pregnancy | 59 | 11.5 (9.5, 16.1) | 30 | 13.5 (9.7, 20.9) | 21 | 12.5 (7.8, 17.6) | 19 | 12.0 (7.4, 15.5) |
| | | Maternal blood in late pregnancy | 78 | 8.4 (6.0, 11.4) | 14 | 9.8 (6.3, 20.0) | 15 | 8.4 (7.2, 14.5) | 9 | 7.3 (5.4, 13.9) |
| | | Maternal blood at delivery | 72 | 5.6 (3.9, 8.1) $^{a}$ | 12 | 6.9 (4.8, 9.8) | 15 | 8.4 (5.5, 13.1) $^{b}$ | 9 | 6.6 (5.5, 9.0) |
| | | Cord blood | 76 | 8.3 (6.3, 10.7) | 14 | 11.1 (6.6, 13.0) | 15 | 10.2 (8.0, 14.1) | 8 | 8.4 (5.8, 12.2) |
| Methionine Cycle | Methionine, μmol/L | Maternal blood in early pregnancy | 59 | 18.7 (16.4, 22.8) $^{a}$ | 30 | 17.9 (15.9, 21.4) $^{a}$ | 21 | 23.7 (19.4, 29.9) $^{b}$ | 20 | 18.3 (15.5, 25.3) |
| | | Maternal blood in late pregnancy | 78 | 19.0 (16.6, 22.9) | 14 | 19.3 (16.5, 22.6) | 15 | 18.7 (16.7, 22.6) | 9 | 20.9 (18.0, 23.7) |
| | | Maternal blood at delivery | 72 | 20.2 (17.8, 23.4) | 12 | 20.0 (18.1, 26.5) | 15 | 21.6 (18.4, 23.0) | 9 | 19.9 (17.1, 25.4) |
| | | Cord blood | 76 | 29.4 (27.3, 32.2) | 14 | 29.9 (28.1, 33.4) | 15 | 29.9 (29.3, 35.2) | 8 | 33.4 (31.1, 39.1) |
| | SAM, nmol/L | Maternal blood in early pregnancy | 59 | 56.1 (48.6, 64.2) $^{a}$ | 30 | 56.9 (53.0, 62.5) $^{a}$ | 21 | 68.5 (59.3, 79.5) $^{b}$ | 20 | 58.5 (48.2, 71.8) |
| | | Maternal blood in late pregnancy | 78 | 56.3 (48.9, 63.6) | 14 | 60.8 (50.5, 74.0) | 15 | 61.6 (57.0, 70.8) | 9 | 62.9 (54.2, 69.3) |
| | | Maternal blood at delivery | 72 | 58.3 (49.6, 66.9) | 12 | 61.7 (47.9, 70.4) | 15 | 66.1 (55.4, 73.4) | 9 | 58.2 (47.3, 68.4) |
| | | Cord blood | 76 | 112.0 (96.7, 128.3) | 14 | 113.3 (106.0, 130.1) | 15 | 126.5 (105.3, 143.5) | 8 | 122.8 (99.1, 139.1) |
| | SAH, nmol/L | Maternal blood in early pregnancy | 59 | 11.1 (9.0, 13.9) | 30 | 10.2 (9.0, 12.6) $^{a}$ | 21 | 13.4 (11.1, 16.7) $^{b}$ | 20 | 10.8 (9.5, 11.9) |
| | | Maternal blood in late pregnancy | 78 | 12.4 (9.8, 14.0) | 14 | 12.0 (9.1, 14.9) | 15 | 12.6 (11.5, 14.3) | 9 | 12.4 (9.8, 18.0) |
| | | Maternal blood at delivery | 72 | 22.1 (18.6, 28.4) | 12 | 31.3 (17.3, 33.7) | 15 | 23.9 (21.7, 32.8) | 9 | 24.5 (19.8, 38.5) |
| | | Cord blood | 76 | 42.4 (37.3, 50.4) | 14 | 51.6 (41.0, 73.1) | 15 | 50.3 (43.2, 63.5) | 8 | 39.4 (29.8, 47.6) |
| | SAM/SAH | Maternal blood in early pregnancy | 59 | 5.35 (3.97, 6.25) | 30 | 5.56 (4.31, 6.19) | 21 | 5.17 (4.02, 6.70) | 20 | 5.51 (4.57, 6.28) |
| | | Maternal blood in late pregnancy | 78 | 4.76 (3.87, 5.61) | 14 | 4.74 (4.60, 6.68) | 15 | 4.88 (4.36, 6.69) | 9 | 4.80 (3.52, 6.17) |
| | | Maternal blood at delivery | 72 | 2.68 (1.90, 3.41) | 12 | 2.39 (1.50, 3.81) | 15 | 2.59 (2.01, 3.07) | 9 | 2.08 (1.68, 2.95) |
| | | Cord blood | 76 | 2.63 (2.09, 3.25) | 14 | 2.19 (1.62, 3.13) | 15 | 2.83 (1.66, 3.09) | 8 | 2.90 (2.49, 3.45) |
| Transulfuration Pathway | tHcy, μmol/L | Maternal blood in early pregnancy | 59 | 5.73 (4.87, 6.49) $^{a}$ | 30 | 4.96 (4.36, 5.58) $^{b}$ | 21 | 5.68 (4.32, 6.58) | 20 | 5.23 (4.50, 5.72) |
| | | Maternal blood in late pregnancy | 78 | 5.76 (4.93, 7.34) | 14 | 4.76 (4.32, 6.39) | 15 | 5.07 (3.59, 6.31) | 9 | 6.13 (3.91, 7.00) |
| | | Maternal blood at delivery | 72 | 7.46 (6.50, 9.30) $^{a}$ | 12 | 5.91 (4.89, 8.06) | 15 | 5.96 (4.55, 7.62) $^{b}$ | 9 | 5.86 (4.83, 8.57) |
| | | Cord blood | 76 | 6.29 (5.42, 7.89) $^{a}$ | 14 | 5.21 (4.31, 7.01) | 15 | 4.93 (3.88, 5.89) $^{b}$ | 8 | 6.17 (3.99, 7.22) |
| | Cystathionine, nmol/L | Maternal blood in early pregnancy | 59 | 105 (82, 133) | 30 | 92 (73, 116) | 21 | 130 (86, 159) | 20 | 93 (72, 121) |
| | | Maternal blood in late pregnancy | 78 | 218 (178, 288) | 14 | 171 (150, 270) | 15 | 223 (165, 350) | 9 | 213 (176, 256) |
| | | Maternal blood at delivery | 72 | 219 (183, 315) | 12 | 175 (129, 225) | 15 | 213 (156, 358) | 9 | 185 (127, 254) |
| | | Cord blood | 76 | 324 (238, 405) | 14 | 305 (237, 332) | 15 | 335 (287, 490) | 8 | 286 (259, 359) |
| | tCys, μmol/L | Maternal blood in early pregnancy | 59 | 233 (214, 258) | 30 | 235 (211, 255) | 21 | 258 (229, 281) | 20 | 245 (229, 249) |
| | | Maternal blood in late pregnancy | 78 | 210 (198, 222) | 14 | 212 (202, 229) | 15 | 223 (210, 246) | 9 | 238 (211, 240) |
| | | Maternal blood at delivery | 72 | 232 (216, 262) | 12 | 241 (211, 269) | 15 | 254 (224, 265) | 9 | 250 (244, 255) |
| | | Cord blood | 76 | 211 (195, 225) $^{a}$ | 14 | 207 (188, 233) | 15 | 226 (214, 256) $^{b}$ | 8 | 219 (201, 238) |
| | tHcy/tCys×$10^2$ | Maternal blood in early pregnancy | 59 | 2.41 (2.14, 2.88) $^{a}$ | 30 | 2.18 (1.78, 2.42) $^{b}$ | 21 | 2.08 (1.72, 2.51) $^{b}$ | 20 | 2.15 (1.85, 2.36) $^{b}$ |
| | | Maternal blood in late pregnancy | 78 | 2.79 (2.51, 3.38) $^{a}$ | 14 | 2.29 (2.08, 2.80) $^{b}$ | 15 | 2.08 (1.76, 2.54) $^{b}$ | 9 | 2.56 (1.89, 3.11) |
| | | Maternal blood at delivery | 72 | 3.10 (2.82, 3.68) $^{a}$ | 12 | 2.56 (2.27, 3.04) $^{b}$ | 15 | 2.24 (1.93, 2.90) $^{b}$ | 9 | 2.31 (2.12, 3.31) |
| | | Cord blood | 76 | 3.09 (2.70, 3.56) $^{a}$ | 14 | 2.46 (2.30, 3.05) $^{b}$ | 15 | 2.18 (1.73, 2.47) $^{b}$ | 8 | 2.84 (1.72, 3.36) |
| | Taurine, μmol/L | Maternal blood in early pregnancy | 59 | 64.1 (52.2, 90.0) | 30 | 63.7 (54.5, 97.7) | 21 | 84.5 (58.0, 106.5) | 20 | 80.5 (54.9, 103.2) |
| | | Maternal blood in late pregnancy | 78 | 61.1 (49.9, 78.8) | 14 | 61.0 (51.0, 84.9) | 15 | 53.4 (42.1, 68.6) | 9 | 68.8 (49.1, 77.7) |
| | | Maternal blood at delivery | 72 | 72.0 (49.7, 105.3) | 12 | 67.7 (55.0, 82.0) | 15 | 80.0 (36.7, 117.5) | 9 | 80.9 (74.1, 111.5) |
| | | Cord blood | 76 | 183.3 (148.9, 226.1) | 14 | 195.0 (139.5, 268.6) | 15 | 188.0 (124.0, 206.0) | 8 | 173.0 (141.6, 223.0) |
| Amino Acid | Serine, μmol/L | Maternal blood in early pregnancy | 59 | 100 (89, 113) | 30 | 92 (82, 107) | 21 | 102 (96, 113) | 20 | 92 (76, 102) |
| | | Maternal blood in late pregnancy | 78 | 104 (91, 117) | 14 | 103 (95, 111) | 15 | 103 (91, 110) | 9 | 102 (89, 111) |
| | | Maternal blood at delivery | 72 | 116 (101, 130) | 12 | 113 (98, 137) | 15 | 110 (97, 120) | 9 | 102 (95, 136) |
| | | Cord blood | 76 | 153 (142, 164) | 14 | 154 (135, 170) | 15 | 160 (138, 175) | 8 | 154 (139, 176) |
| | Glycine, μmol/L | Maternal blood in early pregnancy | 59 | 152 (131, 167) $^{a}$ | 30 | 150 (134, 168) | 21 | 180 (147, 209) $^{b}$ | 20 | 149 (141, 181) |
| | | Maternal blood in late pregnancy | 78 | 145 (127, 171) | 14 | 145 (126, 164) | 15 | 157 (151, 179) | 9 | 152 (140, 183) |
| | | Maternal blood at delivery | 72 | 172 (139, 205) | 12 | 184 (148, 229) | 15 | 182 (151, 211) | 9 | 166 (147, 210) |
| | | Cord blood | 76 | 256 (235, 275) | 14 | 250 (225, 287) | 15 | 274 (246, 300) | 8 | 268 (244, 281) |
| | Serine/Glycine | Maternal blood in early pregnancy | 59 | 0.681 (0.554, 0.756) $^{a}$ | 30 | 0.622 (0.562, 0.687) | 21 | 0.600 (0.507, 0.702) | 20 | 0.576 (0.531, 0.651) $^{b}$ |
| | | Maternal blood in late pregnancy | 78 | 0.701 (0.602, 0.806) | 14 | 0.696 (0.665, 0.760) | 15 | 0.624 (0.572, 0.738) | 9 | 0.640 (0.568, 0.689) |
| | | Maternal blood at delivery | 72 | 0.663 (0.580, 0.758) | 12 | 0.644 (0.560, 0.726) | 15 | 0.617 (0.499, 0.738) | 9 | 0.647 (0.578, 0.719) |
| | | Cord blood | 76 | 0.605 (0.554, 0.654) | 14 | 0.609 (0.539, 0.651) | 15 | 0.603 (0.551, 0.617) | 8 | 0.576 (0.505, 0.682) |
| Co-factor | Riboflavin, nmol/L | Maternal blood in early pregnancy | 59 | 7.84 (0.00, 14.30) $^{a}$ | 30 | 9.68 (3.94, 24.25) | 21 | 21.95 (12.51, 34.85) $^{b}$ | 20 | 6.20 (0.61, 15.64) $^{b}$ |
| | | Maternal blood in late pregnancy | 78 | 5.59 (0.00, 12.53) $^{a}$ | 14 | 7.57 (2.76, 15.99) | 15 | 24.55 (5.55, 60.75) $^{b}$ | 9 | 8.65 (4.11, 21.48) |
| | | Maternal blood at delivery | 72 | 4.95 (0.00, 12.34) | 12 | 8.26 (5.47, 13.53) | 15 | 13.50 (7.00, 24.40) | 9 | 9.91 (7.55, 22.25) |
| | | Cord blood | 76 | 50.85 (32.00, 69.58) | 14 | 59.90 (35.83, 94.99) | 15 | 56.50 (33.90, 121.00) | 8 | 79.70 (41.94, 136.01) |
| | Pyridoxamine, nmol/L | Maternal blood in early pregnancy | 59 | 0.218 (0.168, 0.276) | 30 | 0.227 (0.191, 0.259) | 21 | 0.205 (0.168, 0.286) | 20 | 0.223 (0.159, 0.298) |
| | | Maternal blood in late pregnancy | 78 | 0.203 (0.174, 0.267) | 14 | 0.241 (0.190, 0.287) | 15 | 0.226 (0.170, 0.262) | 9 | 0.248 (0.197, 0.380) |
| | | Maternal blood at delivery | 72 | 0.228 (0.187, 0.302) | 12 | 0.257 (0.202, 0.344) | 15 | 0.246 (0.204, 0.288) | 9 | 0.261 (0.233, 0.342) |
| | | Cord blood | 76 | 0.287 (0.251, 0.409) | 14 | 0.399 (0.332, 0.464) | 15 | 0.349 (0.269, 0.478) | 8 | 0.409 (0.329, 0.469) |
| | Pyridoxine, nmol/L | Maternal blood in early pregnancy | 59 | 0.108 (0.070, 0.180) | 30 | 0.128 (0.095, 0.203) | 21 | 0.138 (0.101, 0.187) | 20 | 0.160 (0.092, 0.239) |
| | | Maternal blood in late pregnancy | 78 | 0.121 (0.066, 0.191) | 14 | 0.160 (0.095, 0.217) | 15 | 0.111 (0.074, 0.231) | 9 | 0.139 (0.086, 0.172) |
| | | Maternal blood at delivery | 72 | 0.122 (0.071, 0.166) | 12 | 0.098 (0.080, 0.170) | 15 | 0.148 (0.103, 0.172) | 9 | 0.053 (0.045, 0.333) |
| | | Cord blood | 76 | 0.191 (0.117, 0.302) | 14 | 0.351 (0.136, 0.426) | 15 | 0.207 (0.153, 0.695) | 8 | 0.231 (0.149, 0.313) |

Variables are expressed as median (25th, 75th percentile).

* Maternal blood at delivery and cord blood were stratified based on late pregnancy criteria.

The difference in serum concentrations of OCM-related metabolites between the FA non-users and the FA users was tested using the Mann-Whitney U test.

Differences in serum OCM-related metabolite concentrations across FA intake categories were evaluated using the Steel-Dwass test for all pairwise comparisons.

Values not sharing a common superscript (a, b, c, d) differ significantly ($p < 0.05$).
